# Conserved C-terminal residues adjacent to the canonical PDX1 homeodomain are required for chromatin occupancy and pancreas development

**DOI:** 10.1242/dev.205842

**Published:** 2026-10-20

**Authors:** Anthony S. Wokasch, Muhammad Shamoon, Courtney Campbell, Tianqi Wu, Jennifer D. Fuhr, Anna Gilbert, Thomas Mehaffey, Carter M. Barnes, Neha Ahuja, Morgan A. Gingerich, Jie Zhu, Paul Gadue, Ondine Cleaver, Hassane Mchaourab, Doris A. Stoffers, Scott A. Soleimanpour, Maureen Gannon

**Affiliations:** ^1^ Department of Cell & Developmental Biology, Vanderbilt University, Nashville, TN 37240, USA; ^2^ Department of Internal Medicine, Division of Metabolism, Endocrinology, and Diabetes, University of Michigan, Ann Arbor, MI 48109, USA; ^3^ Department of Molecular Physiology & Biophysics, Vanderbilt University, Nashville, TN 37232, USA; ^4^ Department of Medicine, Division of Diabetes, Endocrinology and Metabolism, Vanderbilt University Medical Center, Nashville, TN 37232, USA; ^5^ Department of Biochemistry, Vanderbilt University, Nashville, TN 37232, USA; ^6^ Department of Biological Sciences, Vanderbilt University, Nashville, TN 37240, USA; ^7^ Department of Molecular Biology, University of Texas Southwestern Medical Center, Dallas, TX 75390, USA; ^8^ Department of Pathology and Laboratory Medicine, The Children's Hospital of Philadelphia, Philadelphia, PA 19105, USA; ^9^ Center for AI in Protein Dynamics, Vanderbilt University, Nashville, TN 37232, USA; ^10^ Department of Medicine, Institute for Diabetes, Obesity, and Metabolism, Perelman School of Medicine, University of Pennsylvania, Philadelphia, PA 19104, USA; ^11^ Department of Molecular and Integrative Physiology, University of Michigan, Ann Arbor, MI 48109, USA; ^12^ VA Ann Arbor Healthcare System, Ann Arbor, MI 48105, USA

**Keywords:** Pancreas development, Transcription factor, Endocrine specification, Homeodomain, CUT&RUN

## Abstract

Essential roles for the PDX1 transcription factor in pancreatic development and β-cell function are well known, yet the structure-function relationships of domains within its C-terminus remain incompletely defined. We identified a focal intrinsically disordered region (IDR) spanning residues 207-223 of the PDX1 C-terminus and generated mice harboring deletion of this region (Pdx1Δ207). Despite normal protein levels, Pdx1Δ207 homozygous embryos exhibit severe pancreas hypoplasia by embryonic day (E)11.5, with particular impairment in endocrine differentiation. Although Pdx1Δ207 localization was predominantly nuclear in β cells, further biochemical analyses revealed loss of chromatin occupancy at PDX1 target genes and decreased transcriptional activation of the *Ins2* promoter. Truncation of the C-terminus beginning at residue 211 had no effect on target gene occupancy, localizing the functional requirement to highly conserved residues 207-210. Interestingly, structural predictions suggest that residues 207-210 incorporate into an extended helical structure immediately adjacent to the PDX1 homeodomain, suggesting potential conformational transitions within this IDR. Taken together, these four conserved residues are required for optimal PDX1 chromatin occupancy and pancreas organogenesis.

## Teaser

**Summary:** Four highly conserved amino acids directly C-terminal to the PDX1 homeodomain are essential for chromatin occupancy at PDX1 target loci, and required for both pancreas organogenesis and β-cell development.

## INTRODUCTION

Developmental mechanisms that affect the mass of pancreatic insulin-secreting β cells at birth can influence susceptibility to diabetes later in life (Gregg et al., 2012; Linnemann et al., 2014). Monogenic forms of diabetes caused by variations in a single gene typically impact the function of transcriptional regulators involved in pancreatic endocrine development. One such gene is pancreas and duodenal homeobox 1 (*PDX1*), a homeodomain transcription factor mutated in individuals with MODY4, a form of monogenic diabetes (Stoffers et al., 1997a). PDX1 is expressed in the pre-pancreatic endoderm of the mouse beginning at E8.5 (Ahlgren et al., 1996). During early embryogenesis, PDX1 is expressed within multipotent pancreatic progenitor cells (MPCs), but becomes restricted mainly to β cells, δ cells and subsets of acinar cells postnatally (Wu et al., 1997). In humans and mice, global loss of *Pdx1* results in pancreas agenesis, leading to diabetes soon after birth (Jonsson et al., 1994; Offield et al., 1996; Stoffers et al., 1997b). Lineage tracing studies in mice have shown that PDX1-positive MPCs give rise to all pancreatic epithelial cell lineages (Gu et al., 2003). PDX1 also plays crucial roles during endocrine specification by directly activating the gene neurogenin 3 (*Ngn3*) (Oliver-Krasinski et al., 2009), as well as β-cell development by activating genes involved in β-cell identity and function (Gao et al., 2014).

PDX1 belongs to the family of homeodomain-containing transcription factors, which play fundamental roles in numerous developmental processes. PDX1 contains a central homeodomain characterized by a highly conserved helix-turn-helix DNA-binding domain spanning residues 146-206 (Banerjee-Basu and Baxevanis, 2001). X-ray crystallography and nuclear magnetic resonance (NMR) studies of PDX1 show that its 60-residue homeodomain contains three α-helices. The first two are antiparallel and position the third helix, the DNA recognition helix, to fit into the major groove, where it specifically contacts ‘TAAT’ A-box motifs (Bastidas and Showalter, 2013; Longo et al., 2007). Residues within the DNA recognition helix are among the most highly conserved across homeodomain proteins, underscoring their functional importance (Banerjee-Basu and Baxevanis, 2001). Outside the homeodomain, NMR analyses indicate that the PDX1 C-terminus lacks defined secondary structure, classifying it as an intrinsically disordered region (IDR) (Cook et al., 2019; Sahu et al., 2014).

IDRs are functional protein regions that, rather than adopting a fixed three-dimensional shape, remain conformationally flexible, a property that enables them to support diverse molecular roles including localization, flexibility/specificity for DNA binding, protein-protein interactions and transcriptional activation (Tompa, 2012; Uversky, 2019). Consistent with this, the PDX1 N-terminus mediates transactivation through direct interaction with co-factors (Stanojevic et al., 2004; Swift et al., 1998). However, the function of the PDX1 C-terminus beyond residue 206 remains incompletely defined. In mice, homozygous truncation of the C-terminus at Ser211 (Pdx1ΔC) severely impairs endocrine development, leading to β-cell dysfunction and hyperglycemia at 3 weeks of age (Oliver-Krasinski et al., 2009). Multiple single nucleotide polymorphisms (SNPs) in the PDX1 C-terminus are also associated with MODY and T2D (Cockburn et al., 2004; Gragnoli et al., 2005; Hani et al., 1999; Weng et al., 2001). Therefore, investigating this domain will offer insights into the etiology and potential interventions for T2D and MODY caused by alterations within this region.

We identified a focal region between residues 207 and 223 of the PDX1 C-terminus predicted to have a high probability for intrinsic disorder. This region lies directly adjacent to the PDX1 homeodomain and contains known human diabetes-associated SNPs (Steinthorsdottir et al., 2014; Weng et al., 2001). To define the function of this focal IDR *in vivo*, we generated mice harboring a deletion of these residues (hereafter, Pdx1Δ207), and compared their developmental phenotype to mice carrying a deletion of the same number of residues shifted downstream, residues 216-232 (hereafter, Pdx1Δ216), and with premature truncation of the PDX1 C-terminus at residue Ser211 (Pdx1ΔC) (Oliver-Krasinski et al., 2009).

Pdx1Δ207 homozygotes exhibit severe pancreas hypoplasia and reduced endocrine differentiation despite normal PDX1 protein levels. Developmental defects, include reduced dorsal and ventral bud volumes, reduced endocrine progenitors, and reduced β-cell differentiation, maturation and proliferation. Pdx1Δ216 embryos showed no observable differences compared to control littermates. Live-cell imaging in the mouse insulinoma 6 (MIN6) β-cell line showed that the 207-223 deletion does not significantly impact nuclear localization. CUT&RUN sequencing and reporter assays revealed that loss of 207-223 broadly disrupts PDX1 chromatin occupancy at target sites and dramatically decreases transcriptional activation. Strikingly, computational structural modeling with AlphaFold3 (AF3) (Abramson et al., 2024) predicts that residues at positions 207-210 incorporate into an extended helical structure immediately adjacent to the canonical PDX1 homeodomain that is essential for PDX1 function.

## RESULTS

### Prediction of a focal IDR spanning residues 207-223 of the PDX1 C-terminus

To determine whether distinct subregions within the PDX1 C-terminal IDR exhibit an increased probability for intrinsic disorder, we analyzed the full-length mouse and human PDX1 protein sequences using two independent disorder-prediction algorithms, IUPRED3 (Erdős et al., 2021) and PrDOS (Ishida and Kinoshita, 2007). Both algorithms converged on a focal region of predicted high disorder enrichment spanning residues 207-223 of the mouse PDX1 C-terminus, with a corresponding region identified in human PDX1, offset by one residue (Fig. 1A). These findings are also consistent with biophysical studies validating the presence of an IDR in the PDX1 C-terminus (Cook et al., 2019; Sahu et al., 2014). Notably, this focal IDR is positioned at the junction of the highly conserved and structured DNA-binding homeodomain.

**Fig. 1.**
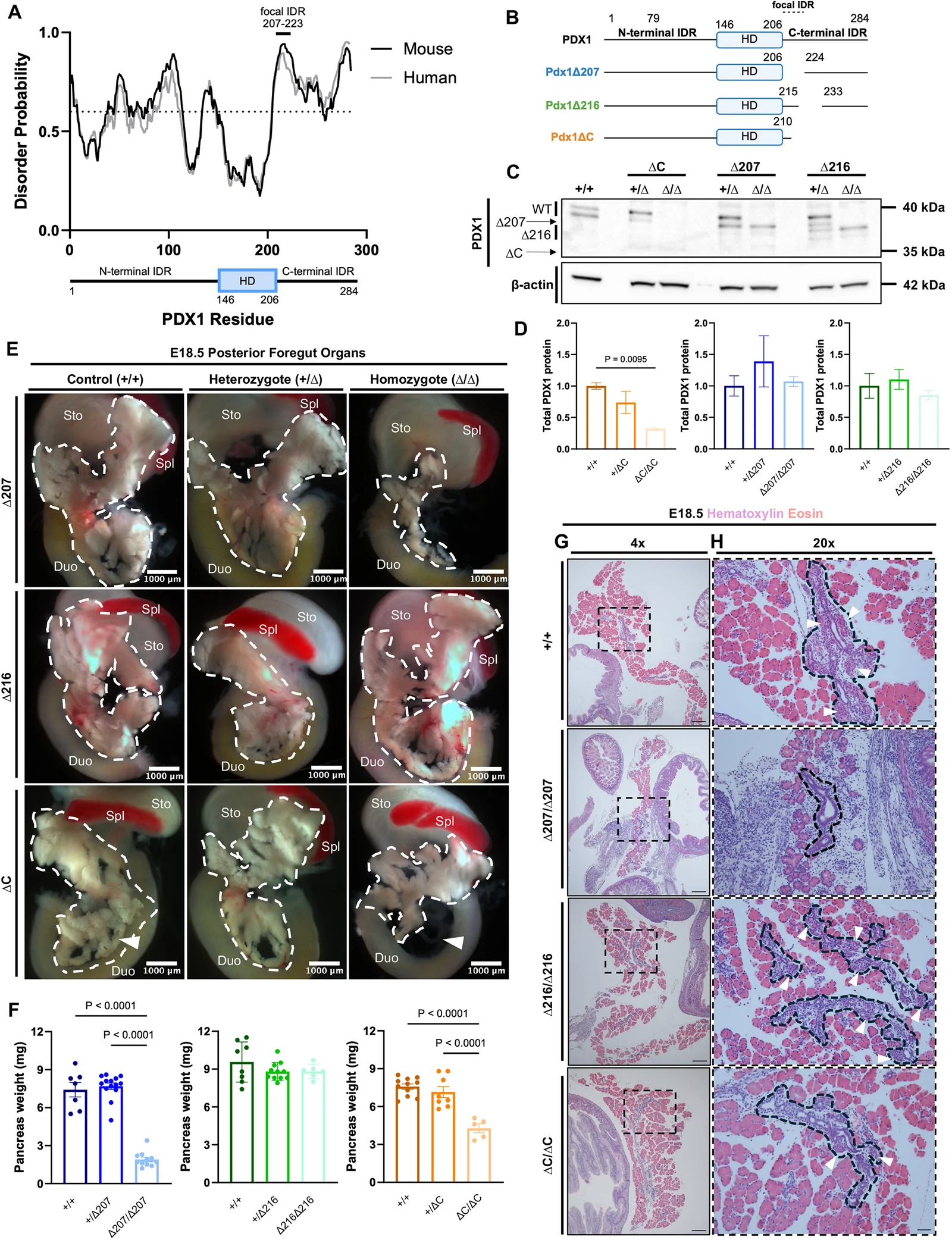
**Deletion of the PDX1 C-terminal focal intrinsically disordered region spanning residues 207-223 causes severe pancreas hypoplasia despite normal PDX1 protein levels.** (A) Mean disorder score from IUPred 3 and PrDOS on mouse (black) and human (gray) PDX1 sequences. Disorder regions identified with a probability threshold of >0.6. (B) PDX1 C-terminal mutant mouse lines. Created in BioRender by Wokasch, A., 2026. https://BioRender.com/ve4fkjb. This figure was sublicensed under CC-BY 4.0 terms. (C) Western blot of PDX1 and β-actin protein extracted from E18.5 whole-pancreas lysates. Representative wild-type control (+/+), heterozygote (+/Δ) and homozygote (Δ/Δ) for each line. Refer to Fig. S1A for complete blots. (D) Densitometric quantification of total PDX1 protein at E18.5 relative to β-actin and normalized to control littermates (*n*=3). (E) Representative images of E18.5 foregut organs. Pancreas tissue is outlined in white. Duo, duodenum; Spl, spleen; Sto, stomach. (F) E18.5 pancreas weight (*n*=5-14). (G,H) Representative tissue sections at E18.5 stained with Hematoxylin and Eosin. Scale bars: 200 μm in G; 50 μm in H. Area outlined in G is magnified in H. (H) The presence of endocrine cell clusters (white arrowheads) surrounding the ductal epithelium (dashed line). *P*-values are displayed (one-way ANOVA with Tukey's). Data are mean±s.e.m.

### Deletion of the PDX1 C-terminal focal IDR causes severe pancreas hypoplasia

To assess the functional significance of the C-terminal focal IDR between residues 207 and 223, we used CRISPR-Cas9 genome editing to delete these residues, thus generating Pdx1Δ207 mutant mice. CRISPR-Cas9 editing also produced an independent line, Pdx1Δ216, harboring an overlapping but distinct 17-residue deletion (216-232) shifted C terminally (Fig. 1B). Since Pdx1Δ216 removes the same number of residues, but does not include the entire focal IDR, we used this line as a control to determine whether developmental defects observed in Pdx1Δ207 are specifically attributable to this deletion rather than to the removal of any stretch of 17-residues within the C-terminus. The previously published Pdx1ΔC mouse line was used as a comparator (Oliver-Krasinski et al., 2009).

To determine whether loss of the PDX1 C-terminal focal IDR has an impact on PDX1 protein levels, posterior foregut organs were dissected at E18.5. Western blot analysis of whole pancreas lysates at E18.5 revealed comparable levels of PDX1 protein in control, Pdx1Δ207 and Pdx1Δ216 homozygotes (Fig. 1C,D; Fig. S1A,B). In contrast, Pdx1ΔC homozygotes exhibited a significant reduction in total PDX1 protein, consistent with the previous report (Fig. 1C,D; Fig. S1A-B) (Oliver-Krasinski et al., 2009).

At E18.5, PDX1 protein is largely restricted to β cells and δ cells, with lower expression in ductal and acinar compartments (Wu et al., 1997). We next assessed spatial PDX1 expression by immunolabelling E18.5 tissue sections for the β-cell marker insulin, acinar cell marker amylase and PDX1 (Fig. S1C-E). Consistent with the western blot analysis, PDX1 protein was readily detectable in insulin^+^ and amylase^+^ cells in pancreata from controls, Pdx1Δ207 and Pdx1Δ216 homozygotes, although there was a marked reduction in the number of insulin^+^ cells observed in Pdx1Δ207 mutants (Fig. S1C,D). Pdx1ΔC homozygotes had noticeably reduced PDX1 intensity within insulin^+^ cells and minimal expression within amylase^+^ cells (Fig. S1E), in agreement with the western blot results.

Pdx1Δ207 homozygotes displayed severe pancreas hypoplasia at E18.5 (75% weight reduction) with defects in pancreas organogenesis evident as early as E15.5, while heterozygotes showed no gross morphological differences or changes in pancreas weight (Fig. 1E,F; Fig. S2A). By contrast, Pdx1Δ216 embryos did not exhibit any differences in pancreas size or weight at E18.5 (Fig. 1E,F). Pdx1ΔC homozygotes displayed milder pancreas hypoplasia, 44% weight reduction at E18.5, and lacked ventral bud-derived regions of the pancreas as early as E15.5 (Fig. 1E,F; Fig. S2A). Although a previous study found no change in pancreas size or weight in mice homozygous for the PDX1 C-terminal deletion at postnatal day P(0) (Oliver-Krasinski et al., 2009), we observed evidence of hypoplasia at both stages (Fig. 1E; Fig. S2A).

Hematoxylin and Eosin staining revealed that Pdx1Δ207 homozygotes displayed swollen cystic ducts lacking observable endocrine cell clusters surrounding the ductal epithelium (Fig. 1G,H; Fig. S2B,C). The swollen main duct of Pdx1Δ207 homozygotes (Fig. S2B,C) was reminiscent of the *Oc1* mutant phenotype in which monocilia formation is impaired (Zhang et al., 2009). Immunostaining for the cilia marker ARL13B (ADP-ribosylation factor-like 13B) showed an apparent reduction in ARL13B-positive ductal cells in Pdx1Δ207 homozygous embryos (Fig. S2D). Pdx1Δ216 homozygotes showed no noticeable morphological differences (Fig. 1G,H). By comparison, Pdx1ΔC homozygotes exhibited loss of ventrally derived pancreas tissue and a reduced number of endocrine cell clusters at both stages (Fig. 1G,H; Fig. S2B,C). These data suggest that deletion of the focal IDR causes more severe pancreatic developmental defects than the C-terminal truncation.

### The PDX1 C-terminal focal IDR is required for primary wave pancreatic bud development

The pancreas first emerges from the posterior foregut endoderm epithelium as two distinct dorsal and ventral buds, which fuse to create one single organ. Pancreatic bud size during the primary wave of development (E9.5-E12.5) dictates the ultimate size of the pancreas (Stanger et al., 2007). To assess early pancreatic bud volume, E11.5 posterior foregut organs of Pdx1Δ207 and Pdx1ΔC embryos were immunolabeled for the epithelial marker E-cadherin (ECAD) and the apical lumen marker mucin 1 (MUC1) (Fig. 2A; Movie 1). Pdx1Δ207 homozygotes showed significant reduction in bud volume with ventral bud outgrowth severely impacted (97%); the bud volume of heterozygotes remained unaffected (Fig. 2A-E; Movie 2). Pdx1ΔC homozygotes exhibited 45% and 75% reductions in dorsal and ventral bud volume, respectively, while heterozygotes only displayed defects in ventral bud volume with no noticeable impact on the dorsal bud (Fig. 2A-E; Movie 3).

**Fig. 2.**
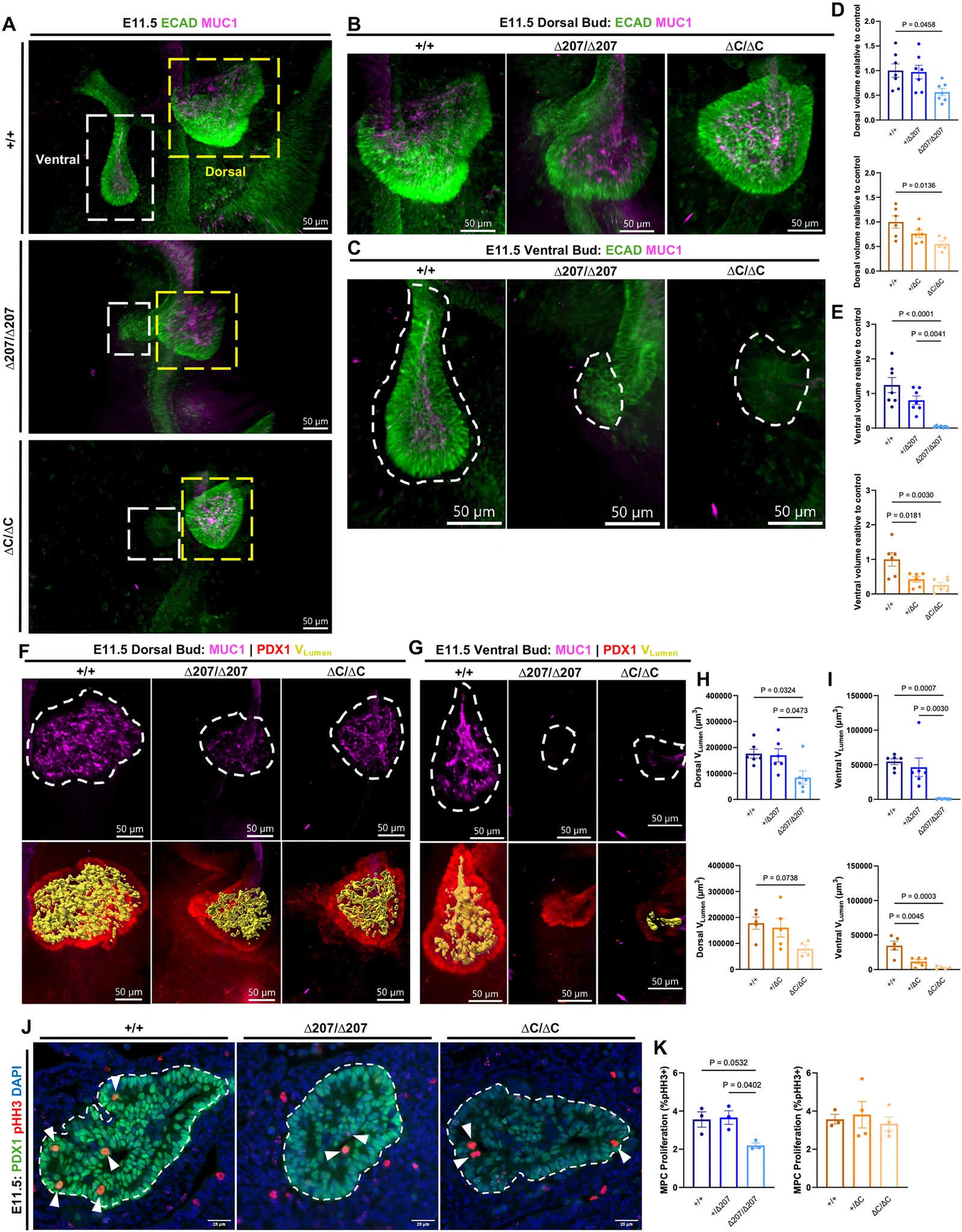
**The PDX1 C-terminal focal intrinsically disordered region is required for primary wave pancreatic bud development and expansion.** (A) Imaris max projections of E11.5 whole-mount embryonic posterior foregut organs immunolabeled for E-cadherin (ECAD; green) and mucin 1 (MUC1; magenta). Dorsal (yellow outlined area) and ventral (white outlined area) pancreatic buds. (B,C) Imaris max projections of E11.5 segmented dorsal (B) and ventral (C) buds. (D,E) Quantification of E11.5 dorsal (D) and ventral (E) bud volume relative to control embryos (*n*=5-7). (F,G) Imaris max projections of mucin 1 (MUC1; magenta) and PDX1 (red), and the surface rendering of the total luminal volume (V_Lumen_: yellow) within the dorsal (F) and ventral (G) buds at E11.5. (H,I) Quantification of E11.5 dorsal (H) and ventral (I) bud V_Lumen_ (*n*=4-6). (J) E11.5 sections immunolabeled for PDX1 (green) and phosphohistone H3 (pHH3; red). White arrowheads represent proliferating MPCs. (K) Quantification of % MPC proliferation. Each dot represents a single embryo (*n*=3 or 4) (every other tissue section containing pancreas bud was quantified). *P*-values are displayed (one-way ANOVA with Tukey's). Data are mean±s.e.m.

A key step in pancreatic morphogenesis is the formation of an epithelial plexus that later remodels into a complex ductal tree. At E11.5, this luminal network is just emerging as isolated microlumens that coalesce later in development (Pictet et al., 1972). To assess lumen network formation, E11.5 posterior foregut organs were immunolabelled for PDX1 and MUC1. Pdx1Δ207 homozygotes showed significant reduction in dorsal bud lumen volume (V_Lumen_), with minimal epithelial lumens in the ventral bud, whereas Pdx1Δ207 heterozygotes were unchanged compared to controls (Fig. 2F-I). Pdx1ΔC homozygotes displayed similar defects, while Pdx1ΔC heterozygotes displayed only luminal defects in the ventral bud (Fig. 2I). These results indicate that the PDX1 C-terminal focal IDR is required for early pancreatic bud development and concomitant epithelial lumen morphogenesis.

### The PDX1 C-terminal focal IDR is required for pancreatic bud expansion but not primary wave endocrine cells

We next asked whether the marked reduction in pancreatic bud size reflected impaired MPC proliferation or alterations in primary wave glucagon^+^ endocrine cells, the two major cell populations within the E11.5 dorsal bud. Immunolabelling for the MPC marker PDX1 and the mitotic marker phosphohistone H3 (pHH3) revealed that Pdx1Δ207 homozygotes displayed a significant reduction (38%) in the percentage of proliferating PDX1^+^ MPCs within the dorsal bud (Fig. 2J,K). PDX1 protein intensity in these mutants appeared comparable to controls (Fig. 2J; Fig. S3A), consistent with our E18.5 western blot and immunolabeling (Fig. 1C,D; Fig. S1A-C). Interestingly, although Pdx1ΔC homozygotes displayed reduced dorsal bud volume (Fig. 2B,C), they showed no discernable changes in the percentage of proliferating PDX1^+^ MPCs within the dorsal pancreatic bud (Fig. 2J,K). However, consistent with our western blots and immunolabelling analyses, PDX1 protein expression was visibly reduced compared to controls (Fig. 2J; Fig. S3A). We observed a modest increase in the number of glucagon^+^ cells normalized to bud area in both Pdx1Δ207 and Pdx1ΔC homozygotes (Fig. S3A,B); however, this likely reflects the smaller dorsal bud size of these embryos rather than an increase in the primary wave of endocrine cell population (Fig. 2B,C).

### Deletion of the PDX1 C-terminal focal IDR impairs ductal morphogenesis

During the secondary wave, isolated microlumens connect with a central main duct to create a complex ductal plexus structure, thereafter undergoing remodeling to form a mature branched ductal tree (Hogan, 1999; Puri and Hebrok, 2007; Villasenor et al., 2010). Since Pdx1Δ207 and Pdx1ΔC homozygotes had defects in lumen formation at E11.5 (Fig. 2F-I), we evaluated the complexity of the ductal network at E15.5. We performed whole-mount immunolabeling for MUC1 to generate a 3D skeleton of the luminal network using the skeletonize plugin in FIJI (Fig. 3A,B), ensuring that the reconstructed network adequately recapitulated MUC1+ luminal structures (Fig. S3C) (Darrigrand et al., 2024).

**Fig. 3.**
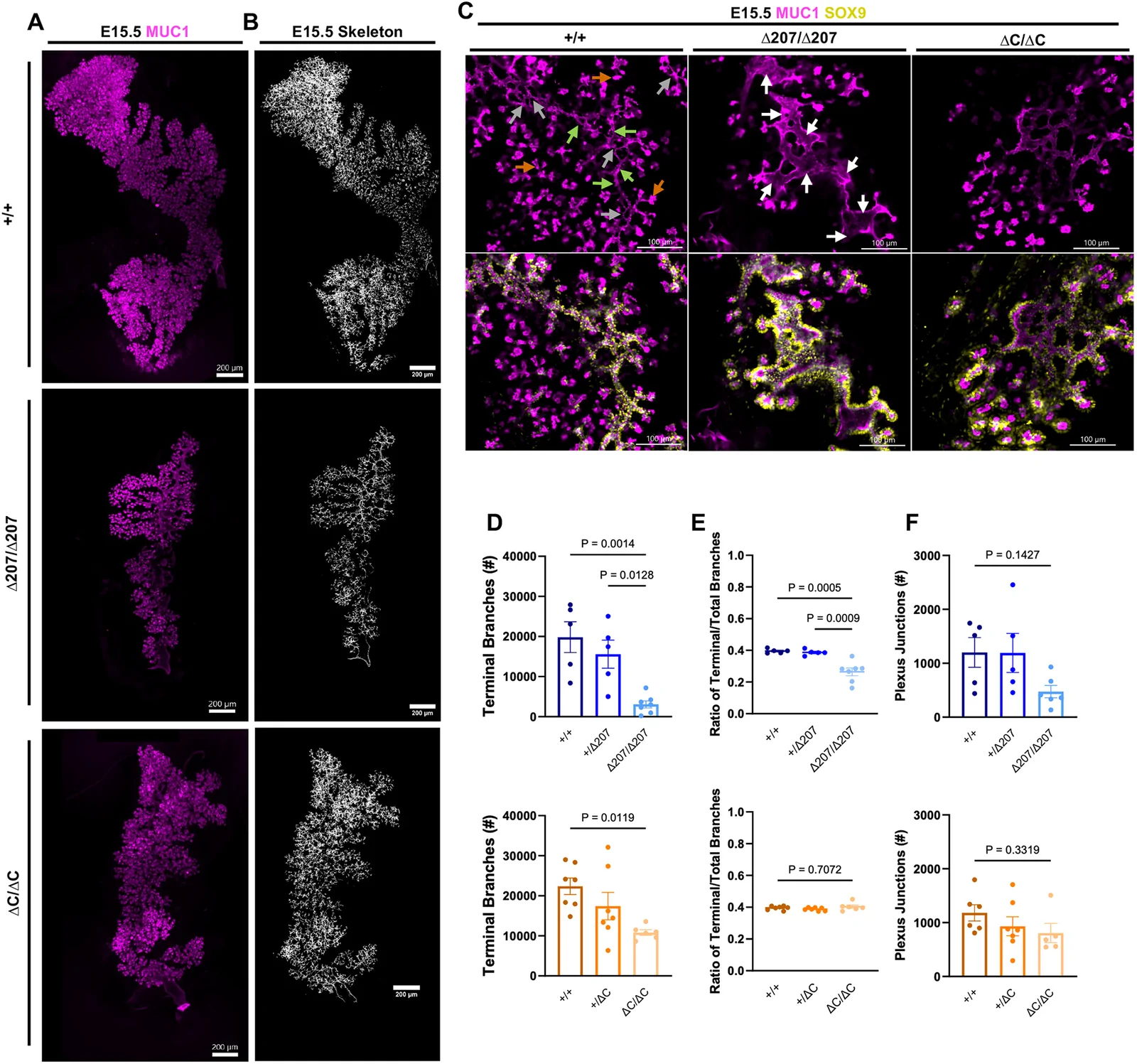
**Deletion of the PDX1 C-terminal focal intrinsically disordered region impairs ductal morphogenesis.** (A) Representative Imaris max projections of E15.5 whole-mount pancreata immunolabeled for mucin 1 (MUC1; magenta). (B) FIJI max projections of E15.5 pancreas skeleton based on MUC1 expression. (C) Imaris slices of E15.5 whole-mount pancreata immunolabeled for MUC1 (magenta) and SOX9 (yellow). Dark-orange arrows indicate branch endpoint, green arrows indicate branches, white arrows indicate swollen lumens and gray arrows indicate plexus junction. Scale bars: 100 μm. (D-F) Quantification of pancreatic ductal network formation at E15.5: total number of terminal branches (D), ratio of terminal branches to total branches (E) and ductal complexity based on the total number of plexus junctions (F) (*n*=5-7). *P*-values are displayed (one-way ANOVA with Tukey's). Data are mean±s.e.m.

Pdx1Δ207 homozygotes exhibited an 85% reduction in the number of terminal branches in the ductal network compared to controls (Fig. 3C,D). To determine whether this reduction simply reflected an overall decrease in ductal network size, we normalized terminal branches to the total number of branches within the network (Fig. 3E). Even after normalization, the ratio of terminal branches remained significantly reduced in Pdx1Δ207 homozygotes (Fig. 3E), demonstrating a disproportionate loss of terminal branches within the network. In contrast, Pdx1ΔC homozygotes exhibited a 52% reduction in the number of terminal branches within the ductal network (Fig. 3D), which was resolved once normalized to bud size (Fig. 3E), indicating that in Pdx1ΔC homozygotes, the decrease in terminal branches reflected a proportionate reduction in the ductal compartment to the overall organ size rather than a specific defect in epithelial morphogenesis.

Because formation of the ductal tree depends on epithelial remodeling during the transient plexus state, we also quantified plexus junctions to determine alterations in network complexity. A plexus junction was defined as a multicellular branch point corresponding to nodes with three or four connecting segments (Fig. 3C). Pdx1Δ207 homozygotes showed strong trends toward reduced plexus junctions compared to controls (Fig. 3C,F) and displayed swollen lumens reminiscent of those reported in *Pdx1*-null embryos (Fig. 3C, white arrows) (Marty-Santos and Cleaver, 2016). These embryos also showed an apparent increase in SOX9^+^ nuclei within the ductal epithelium and acinar cell clusters extending out to the branch tips (Fig. 3C). By contrast, the number of plexus junctions remained unchanged within Pdx1ΔC homozygotes. Neither heterozygote exhibited significant alterations in any of the ductal parameters analyzed (Fig. 3D-F).

### Deletion of the PDX1 C-terminal focal IDR impairs second wave endocrine progenitor specification

The PDX1 C-terminus plays a crucial role in endocrine lineage specification by activating expression of the transcription factor *Ngn3* within bipotential endocrine-ductal progenitor cells (Henley et al., 2016; Oliver-Krasinski et al., 2009). To determine whether the PDX1 C-terminal focal IDR is required for endocrine progenitor specification, we immunolabelled tissue sections for NGN3 and SOX9 to assess cell fate choice. Given the noted expansion of SOX9^+^ cells observed in Pdx1Δ207 homozygotes (Fig. 3C), co-labelling for both markers allowed us to evaluate whether the balance between ductal and endocrine progenitor fates was disrupted.

At E15.5, the peak of second wave endocrine specification, Pdx1Δ207 homozygotes exhibited a 75% reduction in NGN3^+^ nuclei/pancreas area accompanied by a 250% increase in SOX9^+^ nuclei/pancreas area, resulting in a 91% reduction in NGN3^+^:SOX9^+^ ratio (Fig. 4A-D). This result is consistent with a shift toward a ductal/progenitor fate at the expense of endocrine specification. Pdx1Δ207 heterozygotes and Pdx1Δ216 mutants were unaffected (Fig. 4A-D). Pdx1ΔC homozygotes exhibited a significant reduction (68%) in NGN3^+^ nuclei/pancreas area, as expected, and a 69% reduction in NGN3^+^:SOX9^+^ ratio (Fig. 4A-D); however, unlike Pdx1Δ207, the numbers of SOX9^+^ nuclei were unchanged (Fig. 4D), indicating that the endocrine deficit occurred without increased ductal fate allocation. Pdx1ΔC heterozygotes showed a 40% reduction in NGN3^+^:SOX9^+^ nuclei ratio without a significant change in NGN3^+^ nuclei per pancreas area (Fig. 4B,C), revealing a dose-dependent role of the PDX1 C-terminus in endocrine progenitor specification. To determine if endocrine specification was delayed, we examined the number of NGN3^+^ nuclei at E18.5. Similar defects as seen at E15.5 were observed in all mutants; however, Pdx1∆207 heterozygous mutants now displayed a significant reduction in NGN3^+^ nuclei (Fig. 4E).

**Fig. 4.**
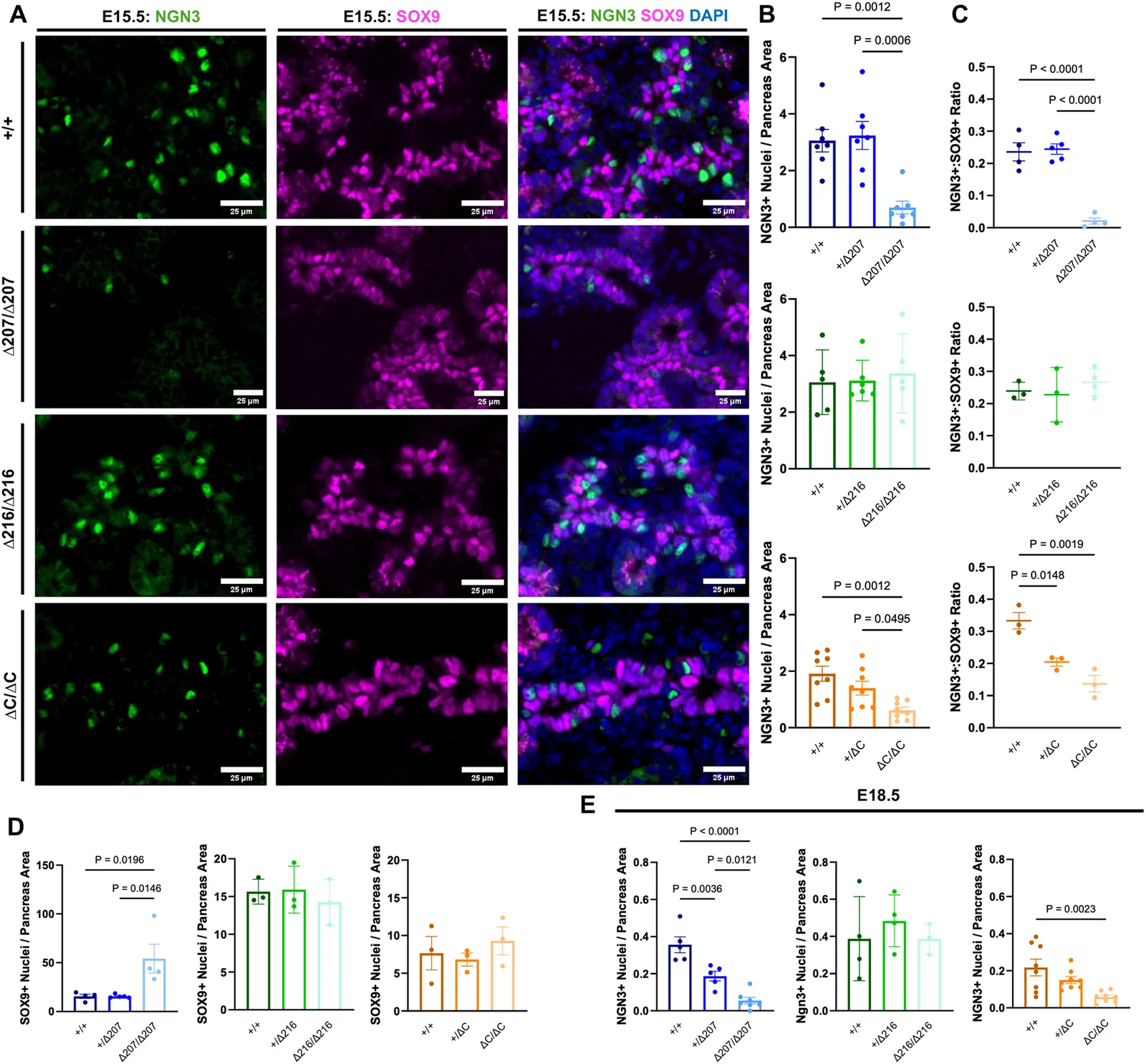
**Deletion of the PDX1 C-terminal focal intrinsically disordered region significantly impairs second wave endocrine progenitor specification.** (A) E15.5 sections immunolabeled for neurogenin 3 (NGN3; green), SOX9 (magenta) and DAPI (blue). (B,C,E) Quantification of (B) NGN3^+^ nuclei/pancreas area (μm^2^×10^−4^) (*n*=7 or 8), (C) NGN3^+^:SOX9^+^ nuclei ratio (*n*=3-5) at E15.5 and (E) E18.5 NGN3^+^ nuclei/pancreas area (μm^2^×10^−4^) (*n*=5-8). (D) SOX9^+^ nuclei per pancreas area (μm^2^×10^−4^) (*n*=3-5). Each dot represents an individual embryo. For each embryo, the mean was calculated from three sections spaced 90 μm (E15.5) or 150 μm (E18.5) apart. *P*-values are displayed (one-way ANOVA with Tukey's). Data are mean±s.e.m.

### The PDX1 C-terminal focal IDR is required for endocrine differentiation, β-cell proliferation and maturation

At late gestation, second-wave endocrine progenitors undergo differentiation, proliferation and maturation to establish mature endocrine cell types required for postnatal glucose homeostasis. To determine how deletion of the PDX1 C-terminal focal IDR influences later stages of endocrine differentiation, we immunolabelled tissue sections for the two main endocrine cell types β (insulin) and α (glucagon) cells at E15.5 and E18.5. Pdx1Δ207 homozygotes displayed ∼90% and 75% reductions in insulin^+^ and glucagon^+^ cells, respectively, at both E15.5 and E18.5 (Fig. 5A-C). The β-cell compartment was disproportionately affected, as evidenced by a reduced INS^+^:GLU^+^ ratio at E18.5 (Fig. S4A). Notably, whereas endocrine cells in control embryos delaminate from the ductal epithelium upon hormone expression, insulin^+^ and glucagon^+^ cells in Pdx1Δ207 homozygotes remained aberrantly embedded within the ductal epithelium (Fig. 5A), revealing a role for this domain in endocrine delamination. Pdx1Δ207 heterozygotes displayed a selective reduction in insulin^+^ cells at both stages with no significant changes in glucagon^+^ cell number or INS^+^:GLU^+^ ratio (Fig. 5B,C; Fig. S4A-C). By contrast, Pdx1Δ216 homozygotes showed no differences in endocrine cell number or INS^+^:GLU^+^ ratio at either stage (Fig. 5A-C; Fig. S4A). In comparison, Pdx1ΔC homozygotes exhibited significant but consistently less severe reductions in endocrine differentiation compared to Pdx1Δ207 homozygotes. Consistent with previous reports (Oliver-Krasinski et al., 2009), insulin^+^ cells were reduced by 70% at E15.5 and 62% at E18.5, while glucagon^+^ cells were reduced by 57% at E15.5 and 22% at E18.5 (Fig. 5A-C), with a similarly reduced INS^+^:GLU^+^ ratio at E18.5 (Fig. S4A). Heterozygotes displayed a transient reduction in both insulin^+^ cells (52%) and glucagon^+^ cells (32%) at E15.5 (Fig. 5B; Fig. S4B) that resolved by E18.5 (Fig. 5C; Fig. S4C), though the INS^+^:GLU^+^ ratio was decreased at the later timepoint (Fig. S4A).

**Fig. 5.**
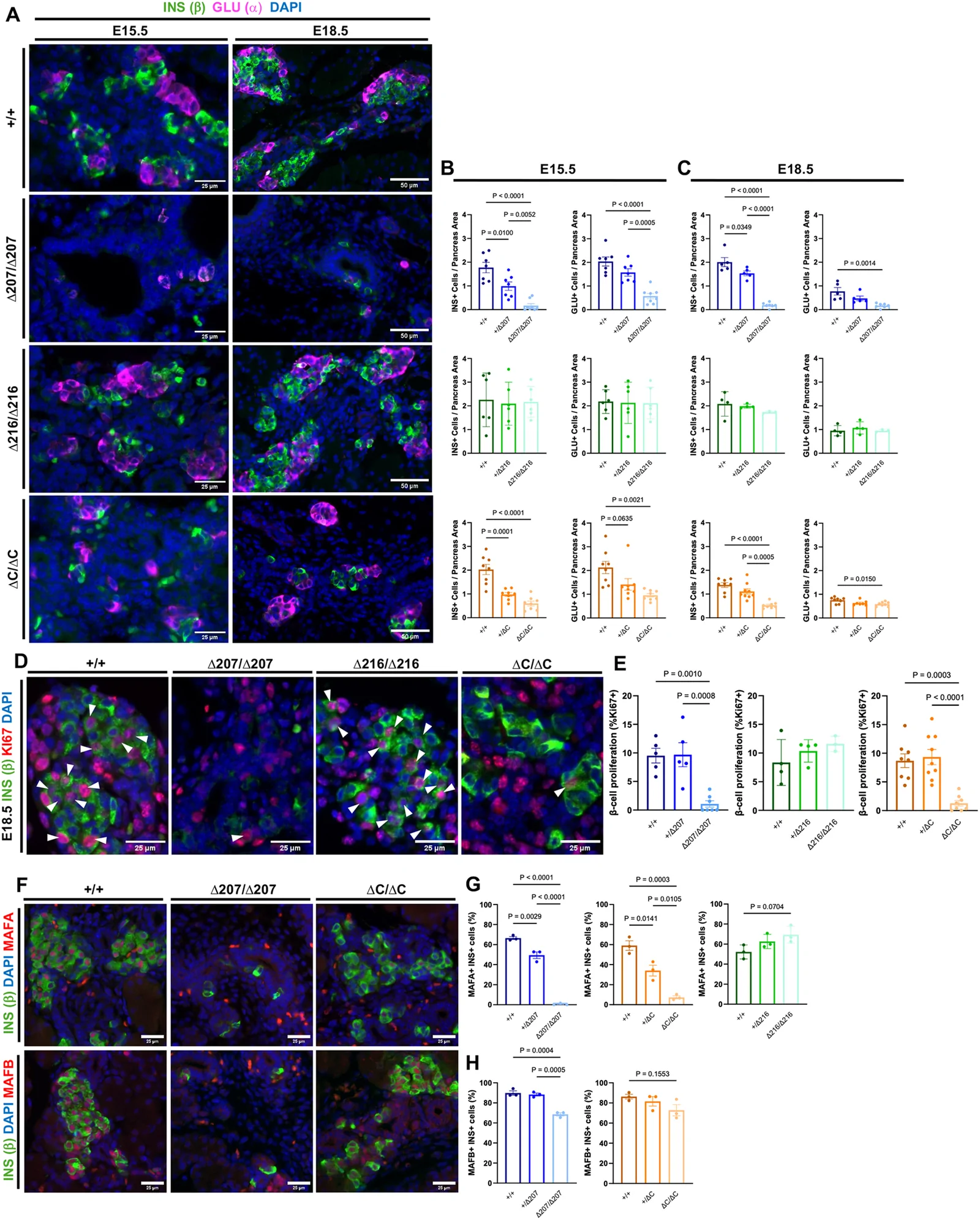
**The PDX1 C-terminal intrinsically disordered region is required for endocrine differentiation, β-cell proliferation and maturation.** (A) E15.5 and E18.5 sections immunolabeled for insulin (INS; green), glucagon (GLU; magenta) and DAPI (blue). (B,C) Quantification of INS^+^ and GLU^+^ cells/area (μm^2^×10^−4^) at E15.5 (B) and E18.5 (C) (*n*=3-8). (D) E18.5 sections immunolabeled for INS (green), KI67 (red) and DAPI (blue). (E) Quantification of β-cell proliferation (%) at E18.5 (*n*=5-8). (F) E18.5 sections immunolabeled for INS (green), with either MAFA or MAFB (red), and DAPI (blue). (G,H) Quantification of the percentage of MAFA+INS+ cells (G) and the percentage of MAFB+INS+ cells (H) at E18.5 (*n*=3). For each embryo, the mean was calculated from three sections spaced 90 μm (E15.5) or 150 μm (E18.5) apart. *P*-values are displayed (one-way ANOVA with Tukey's). Data are mean±s.e.m.

β-Cell proliferation begins at late gestation and continues through perinatal stages (Qiu et al., 2017). Conditional loss of *Pdx1* in developing insulin-expressing cells results in decreased β-cell proliferation with a concomitant increase in α-cell proliferation (Gannon et al., 2008). Given the preferential reduction of the β-cell compartment following loss of the C-terminus and focal IDR, we next examined whether altered proliferation contributes to this phenotype. To assess this, we immunolabeled tissue sections at E18.5 for insulin or glucagon together with the pan-cell cycle marker KI67. At E18.5, Pdx1Δ207 homozygotes exhibited an ∼85% reduction in the percentage of proliferating β cells compared to controls (Fig. 5D,E). Pdx1ΔC homozygotes showed a similar reduction (Fig. 5D,E); however there was no change in α-cell proliferation in either line (Fig. S5B,C). No differences in β- or α-cell proliferation were observed in Pdx1Δ216 homozygous embryos (Fig. 5D,E; Fig. S5A,B).

The large Maf transcription factors MAFB and MAFA are dynamically expressed during β-cell development (Artner et al., 2010; Cyphert et al., 2019). In mice, MAFB is broadly expressed in insulin^+^ cells at E13.5, whereas MAFA is largely restricted to a subset of β cells; by late gestation (∼E18.5), most insulin^+^ cells co-express MAFB and MAFA, marking the normal maturation process of a mouse β cell (Artner et al., 2010). To determine whether loss of the PDX1 C-terminus and focal IDR affects β-cell maturation, we immunolabeled tissue sections at E18.5 for insulin and MAFA or MAFB. At E18.5, Pdx1Δ207 mutants exhibited a profound defect in β-cell maturation, with homozygotes having an almost complete absence of MAFA^+^ insulin^+^ cells, and heterozygotes displaying a significant reduction (Fig. 5F,G). In addition, only Pdx1Δ207 homozygotes showed a significant decrease in the percentage of insulin^+^ cells expressing MAFB (Fig. 5F,H). Pdx1ΔC mutants demonstrated a dose-dependent reduction in MAFA^+^ insulin^+^ cells and no significant change in expression of MAFB (Fig. 5F-H). Pdx1Δ216 homozygotes had no changes in the percentage of MAFA^+^ insulin^+^ cells (Fig. 5G); MAFB expression was not assessed.

### Deletion of the PDX1 C-terminal focal IDR does not prevent nuclear localization

Having established that deletion of the PDX1 C-terminal focal IDR causes severe defects in pancreas development *in vivo* despite normal protein levels, we next sought to define the functional role(s) of the focal IDR. The nuclear localization signal (NLS) of PDX1 has been identified within its homeodomain (residues 198-204), and is required for nuclear import (Moede et al., 1999). Since the C-terminal focal IDR lies in close proximity to the NLS, we assessed whether its deletion impairs nuclear localization. N-terminally mCherry-tagged PDX1 proteins (Fig. S6A) were expressed in MIN6 β cells. Subcellular fractionation revealed no significant reduction in nuclear PDX1 for any of the protein variants (Fig. S6B-D). As PDX1 protein localization is cell-cycle dependent, with PDX1 being nuclear during interphase and cytoplasmic during cell division (Zhu et al., 2021), fractionation experiments alone cannot resolve localization at specific cell-cycle stages. We therefore performed live-cell imaging to assess localization specifically during interphase, when PDX1 is nuclear. All PDX1 C-terminal mutants retained the capacity for nuclear localization (Fig. S6E); however, a very small fraction of Pdx1Δ207 was detected in the cytoplasm during interphase (Fig. S6E, white arrow).

### Deletion of residues 207-223 results in a dramatic reduction of PDX1 chromatin occupancy

Since deletion of the C-terminal focal IDR did not prevent PDX1 nuclear localization, we next sought to determine whether it affects chromatin occupancy. To address this, we performed CUT&RUN sequencing in MIN6 immortalized β cells using antibodies targeting the active chromatin mark H3K4me3 and mCherry to recognize all PDX1 constructs. *E. coli* spike-in normalization (Meers et al., 2019) revealed a strong depletion in Pdx1Δ207 chromatin occupancy (Fig. S7A,B). Genome-wide differential analysis, using DESeq2 (Love et al., 2014), identified a total of 39,457 occupancy sites for wild-type PDX1; 11,933 sites were significantly lost with Pdx1Δ207, including occupancy sites at known PDX1 developmental targets (Fig. 6A). Only six sites were gained that were not present for wild-type PDX1; however, these were at non-biologically relevant targets. By contrast, no significant differential occupancy was detected for either Pdx1ΔC or Pdx1Δ216 relative to wild-type PDX1 (Fig. S7B). While only 11,933 sites met the significance threshold to be considered lost by DESeq2, deepTools3 (Ramírez et al., 2016) visualization of global differences in chromatin occupancy revealed an overall depletion in Pdx1Δ207 signal at all wild-type occupancy sites, with signal intensity indistinguishable from the mCherry-EV control (Fig. 6B; Fig. S7C,D). Notably, Pdx1ΔC and Pdx1Δ216 maintained signal comparable to wild-type at these same regions (Fig. 6B; Fig. S7C).

**Fig. 6.**
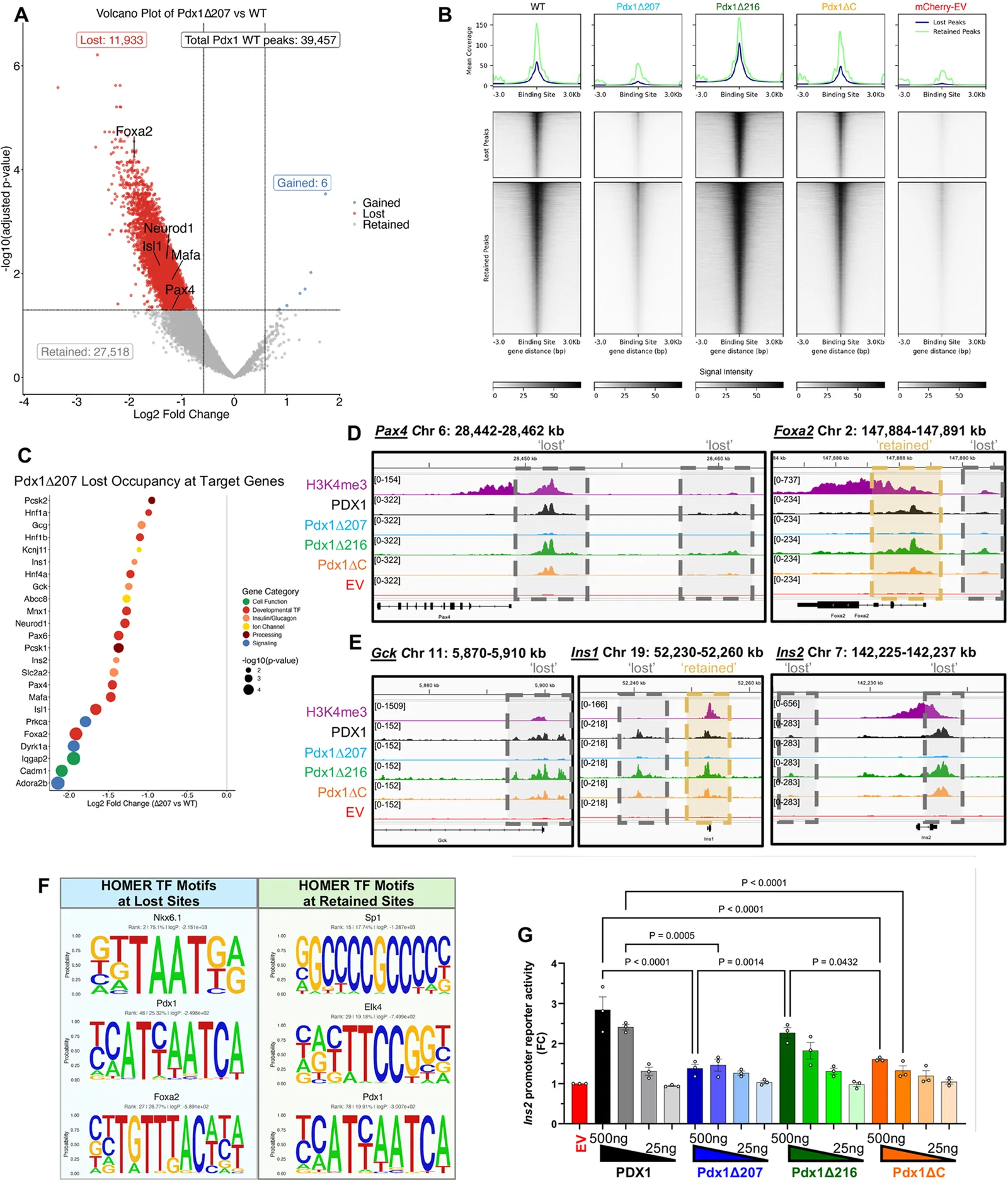
**Deletion of residues 207-223 impairs PDX1 occupancy and transactivation of the *Ins2* promoter.** (A) Volcano plot of differential PDX1 chromatin occupancy in Pdx1Δ207 versus wild-type PDX1 in MIN6 cells. Each point represents a consensus peak colored by significance category. Vertical and horizontal lines indicate significance thresholds (padj<0.05, log2FC≥0.585) (*n*=3). (B) deepTools3 metagene peak-centered heatmap of all lost and retained peaks. (C) Bubble plot of significant Pdx1Δ207 occupancy loss at known pancreatic target genes. (D,E) Representative CUT&RUN-seq IGV tracks at developmental loci (D) and β-cell loci (E). (F) Top-ranked biologically relevant HOMER transcription factor motifs enriched at peaks lost or retained in Pdx1Δ207. (G) *Ins2* promoter luciferase reporter assay depicting activation across PDX1 constructs at multiple doses (500 ng, 250 ng, 125 ng and 25 ng) expressed as fold change relative to mCherry-EV (*n*=3). *P*-values are displayed (one-way ANOVA with Tukey's). Data are mean±s.e.m.

To characterize the distribution of lost and retained peaks, we annotated all Pdx1Δ207-binding sites relative to transcription start sites (TSS) and genomic regions using ChIPseeker (Yu et al., 2015) (Fig. S7E,F). The majority of peaks lost in Pdx1Δ207 were preferentially distributed at distal intergenic and intronic regions >10 kb from the nearest TSS, whereas peaks classified as retained were distributed at the proximal promoter regions ≤3 kb (Fig. S7E,F). This difference in genomic distribution was highly significant (χ^2^ test: *P*-value 2.2×10^−16^). Thus, distal regulatory elements are disproportionately vulnerable to loss upon deletion of residues 207-223.

Filtering for known pancreatic and β-cell genes revealed significant loss of occupancy at multiple genomic regions for key regulatory loci (Fig. 6C; Fig. S7G). Among the most significantly lost sites were those for key transcription factors crucial for pancreatic progenitor specification (*Foxa2, Isl1* and *Mnx1*) and endocrine development (*Neurod1* and *Pax4*) (Fig. 6C). Loss of occupancy also occurred at genes involved in β-cell function (*Ins1/2* and *Gck*), cell signaling (*Dyrk1a* and *Prkca*) and cell survival (*Aatf*) (Fig. 6C). To illustrate the occupancy lost at individual loci, we examined genomic tracks spanning both the distal and promoter regions at two key groups of genes: developmentally relevant (*Foxa2* and *Pax4*) and β-cell specific (*Gck*, *Ins1* and *Ins2)* (Fig. 6D,E). At the distal regulatory regions of each locus, Pdx1Δ207 showed little to no detectable occupancy signal compared to wild-type PDX1, whereas there was variable loss of occupancy at active promoter regions across PDX1 target genes (Fig. 6D,E; Fig. S7G). This pattern is consistent with the DESeq2 analysis in which the promoter regions fell below the adjusted *P*-value threshold (padj<0.05) for statistically significant loss, despite a visibly reduced signal in Pdx1Δ207 tracks (Fig. 6B,D,E). By contrast, Pdx1ΔC and Pdx1Δ216 displayed occupancy comparable to wild-type PDX1 at both distal and proximal regions across all loci examined (Fig. 6D,E).

HOMER motif analysis was used to identify the composition of transcription factor motifs at lost versus retained PDX1 occupancy sites (Heinz et al., 2010). Both peak sets were enriched for homeobox motifs (TAAT), consistent with PDX1 occupying the canonical homeodomain binding site across both categories (Fig. S7H). Lost peaks were additionally enriched for NKX6.1, PDX1, and FOXA2 (Fig. 6F); consistent with their known co-occupancy in primary mouse islets (Ediger et al., 2017). By contrast, retained peaks were selectively enriched for KLF/SP and ETS motifs, notably absent from the lost peak set (Fig. 6F; Fig. S7H), suggesting that PDX1 occupancy at these sites may be resistant to loss of residues 207-223.

### Deletion of residues 207-223 impairs transactivation at the *Ins2* promoter

To assess the transcriptional activation capacity of the different PDX1 mutants, we performed luciferase reporter assays in HEK293T cells using a proximal promoter fragment of *Ins2* containing the well-characterized Area 1 (A1) PDX1 binding motif. Wild-type PDX1 demonstrated robust, dose-dependent transactivation, achieving approximately 3-fold and 2.5-fold increases relative to the mCherry-EV negative control at 500 ng at 250 ng, respectively (Fig. 6G). Pdx1Δ207 showed severely impaired transactivation compared to wild-type PDX1 at 500 ng (*P*<0.0001) and 250 ng (*P*=0.0005) (Fig. 6G), consistent with loss of occupancy at this site (Fig. 7E). Pdx1Δ216 demonstrated a dose-dependent transactivation of the *Ins2* promoter comparable to wild-type PDX1 (Fig. 6G), consistent with the preservation of chromatin occupancy and pancreas development in this mutant. Notably, despite retaining normal chromatin occupancy by CUT&RUN sequencing (Fig. 6D,E), Pdx1ΔC displayed significantly impaired transactivation at the highest doses (*P*<0.0001), with activity similarly reduced to that observed in Pdx1Δ207 (Fig. 6G). Collectively, these data demonstrate a functional requirement for residues 207-223 in optimal chromatin occupancy and transcriptional activation.

**Fig. 7.**
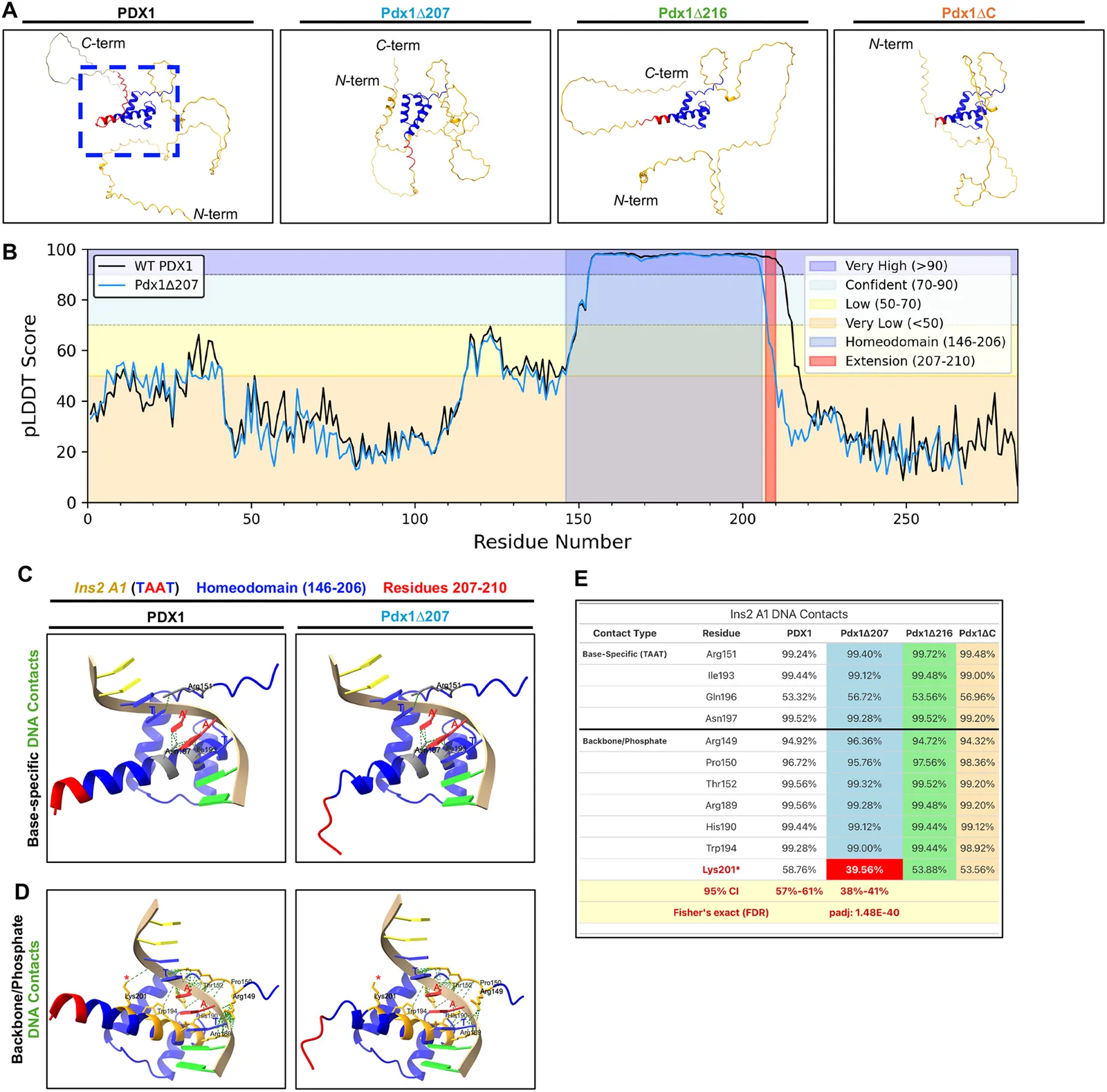
**Residues 207-210 are predicted to form a C-terminal extension of the canonical PDX1 homeodomain but are not predicted to contact DNA.** (A) AF3 highest-confidence models of wild-type PDX1 and each C-terminal mutant in ChimeraX. Homeodomain (146-206, blue) and C-terminal residues of interest (207-232, red) (*n*=2500). (B) AF3 per-residue confidence scores (pLDDT) plotted across full-length wild-type PDX1 (black) and Pdx1Δ207 (blue). (C,D) AF3 highest-confidence models of wild-type PDX1 and Pdx1Δ207 homeodomain (blue) in complex with the *Ins2 A1* TAAT DNA element depicting base-specific contacts (C) and backbone phosphate contacts (D) in green. (E) Protein-*Ins2* DNA contacts, classified as base specific or backbone/phosphate associated analyzed in ChimeraX (*n*=2500).

### Residues 207-210 are predicted to form a C-terminal extension of the canonical PDX1 homeodomain but are not required for direct DNA contact

To investigate the structural basis of the chromatin occupancy defect observed in Pdx1Δ207, we employed AF3 to model the three-dimensional structure of wild-type mouse PDX1 and each C-terminal mutant (Fig. 7A). AF3 predicted with high confidence (average pLDDT=94.7 across Cα atoms) that residues 207-212 form the last two turns of Helix 3 in wild-type PDX1, while the remaining residues (213-223) deleted in Pdx1Δ207 are predicted to be disordered (Fig. 7A). Quantitative analysis of per-residue pLDDT scores confirmed that confidence scores remain consistent between wild-type PDX1 and Pdx1Δ207 across the homeodomain, diverging precisely at residues 207-210 before dropping into the disordered C-terminal domain (Fig. 7B). Because Pdx1Δ207 removes residues 207-223, it truncates the final helix of the PDX1 homeodomain by two turns. In the truncated protein, the newly positioned C-terminal residues (renumbered 207-212) no longer form a helical structure and display low confidence (average pLDDT=52.23) (Fig. 7A). By contrast, Pdx1Δ216 retains residues 207-212 of the wild-type protein (average pLDDT=95.79) and the PDX1 homeodomain final helix is structurally preserved (Fig. 7A). In Pdx1ΔC, truncation at residue 211 removes only the final two residues of the predicted homeodomain third helix extension, and importantly leaves the four residues directly adjacent to the canonical homeodomain intact (Fig. 7A).

Given that AF3 also accurately predicts protein-DNA interactions (Abramson et al., 2024), we next asked whether loss of residues 207-210 was predicted to disrupt direct protein-DNA contacts by modeling each PDX1 protein bound to the *Ins2* A1 promoter (Fig. 7C). Predictions for wild-type PDX1 recapitulated the major base-specific contacts (Arg151, Ile193, Gln196 and Asn197) and backbone/phosphate contacts (Arg149, Pro150, Thr152, Arg189, His190, Trp194 and Lys201) previously identified by X-ray crystallography (Longo et al., 2007) (Fig. 7C-E). All C-terminal mutants, including Pdx1Δ207, retained conservation of every base-specific and backbone contact except one; Pdx1Δ207 alone lost the Lys201 backbone/phosphate contact (Fig. 7D,E). Together, AF3 predicts that residues 207-210 form a structurally important extension of the canonical PDX1 homeodomain, but are not required for direct contact with DNA.

### Residues 207-210 (DKKR) are unique to PDX1 and highly conserved across species

The homeodomain is the most highly conserved region across the homeobox gene family (Banerjee-Basu and Baxevanis, 2001). To assess the conservation of the four residues (207-210) immediately C-terminal to the PDX1 canonical homeodomain (Fig. 8A), we performed multiple sequence alignments (MSA) in Jalview (MAFFT) across representative transcription factors from the Extended HOX, NK, Paired, LIM, POU and atypical homeodomain sub-families, as well as the closely related HOX family (Fig. 8B,C). While residues within the previously defined Helix #3 showed strong conservation across all homeodomain-containing families, as expected, residues 207-210 (DKKR) directly C-terminal to it were poorly conserved across all homeodomain protein families (Fig. 8B-D).

**Fig. 8.**
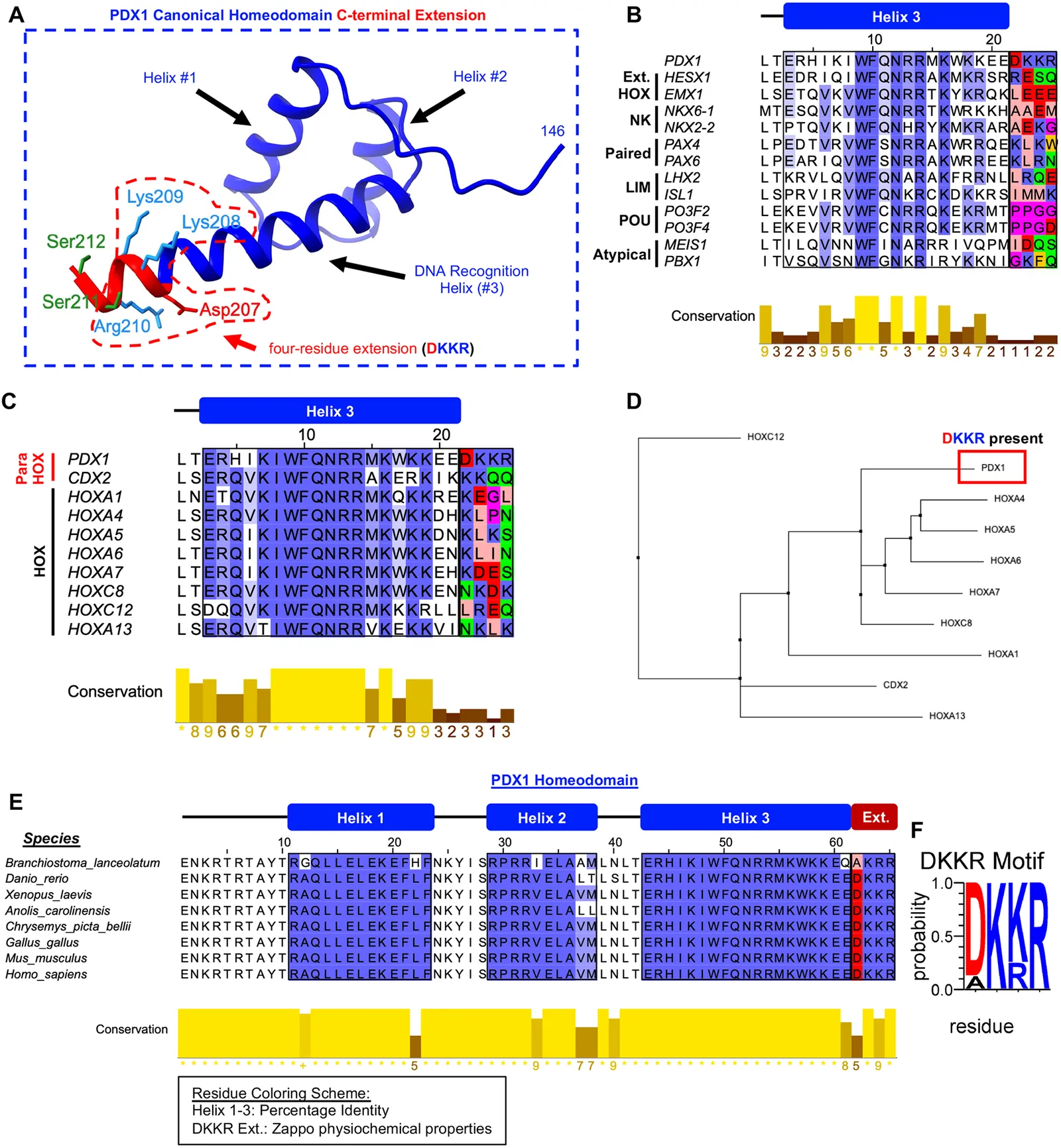
**Residues 207-210 (DKKR) are unique to PDX1 and highly conserved across species.** (A) PDX1 canonical homeodomain (blue) and its predicted C-terminal extension (red) as modeled by AF3. Individual residues comprising the extension are shown and labeled by color: negatively charged, red; positively charged, cyan; polar, green. (B,C) Multiple sequence alignment (MSA) and sequence conservation of PDX1 with the extended homeodomain family of transcription factors (B) and HOX gene cluster (C). (D) BLOSUM62 phylogenetic tree of PDX1 and HOX gene cluster. (E) MSA and sequence conservation of PDX1 orthologs across chordates. (F) Probability of the DKKR residues across chordates.

We next asked whether these PDX1-specific residues are evolutionarily conserved across species. MSA of PDX1 orthologs spanning vertebrate species and the invertebrate chordate amphioxus revealed that, in contrast to the poor conservation of the DKKR residues across the other homeodomain families, this motif is highly conserved across all PDX1 orthologs (Fig. 8E,F). Notably, within the C-terminus, amino acid conservation was largely restricted to the DKKR residues, with only a small number of the remaining C-terminal residues displaying sequence conservation across species (Fig. S8).

## DISCUSSION

PDX1 is required at multiple stages during embryogenesis to ensure the development and proper proportions of all three cellular compartments of the pancreas (Gu et al., 2003; Marty-Santos and Cleaver, 2016). Given the known human diabetes-associated mutations located within the PDX1 C-terminus adjacent to the DNA-binding homeodomain, we interrogated the function of this region using *in vivo* and biochemical approaches. NMR and disorder prediction models classify the 17-residue stretch spanning 207-223 of the PDX1 C-terminus as an IDR, and our analyses reveal that this domain contains a highly conserved four-residue (207-210) region immediately adjacent to the canonical homeodomain that is essential for optimal chromatin occupancy and pancreas development.

Global loss of *Pdx1* in mice and humans results in pancreas agenesis (Jonsson et al., 1994; Offield et al., 1996; Stoffers et al., 1997b), whereas *Pdx1* heterozygosity supports normal pancreas organogenesis but causes a reduction in β-cell and α-cell mass by late gestation, leading to impaired glucose-stimulated insulin secretion later in life (Brissova et al., 2002; Henley et al., 2016; Kropp et al., 2018). Thus, the endocrine compartment is particularly sensitive to PDX1 dosage. In these studies, we show that homozygous deletion of residues 207-223 (Pdx1Δ207) causes defects in early pancreatic bud outgrowth, ductal morphogenesis, endocrine progenitor specification and β-cell development, despite normal levels of Pdx1Δ207 protein. Pdx1Δ207 heterozygotes display impaired β-cell development while retaining normal pancreas organogenesis.

Notably, the ductal morphogenesis defects in Pdx1Δ207 homozygotes included the presence of cystic ducts with visibly reduced primary cilia. Although PDX1 is not expressed in mature ductal cells, it is expressed in the progenitor population that gives rise to the ductal network, where it directly regulates E-cadherin and is essential for ductal morphogenesis (Marty-Santos and Cleaver, 2016). We also observed reduced MUC1 volume and branching defects in Pdx1Δ207 homozygotes, consistent with an early disruption in ductal network development. These structural defects are notable in light of the established role of PDX1 in maintaining acinar cell identity, and preventing acinar-to-ductal metaplasia, a process that can progress to pancreatic intraepithelial neoplasia (PanIN) and pancreatic ductal adenocarcinoma (PDAC) (Roy et al., 2016).

The pancreatic phenotype of Pdx1Δ207 homozygotes was unexpectedly more severe than that of the C-terminal truncation beginning at residue 211 (Pdx1ΔC). The differential effects of these deletions on protein levels may reflect C-terminal-mediated protein-protein interactions. The E3 ubiquitin ligase substrate adapter, speckle-type POZ protein (SPOP), regulates proteasomal turnover of PDX1 through interaction with two C-terminal binding motifs (residues 224-236 and 265-275) (Claiborn et al., 2010; Liu et al., 2006; Usher et al., 2021). Loss of both SPOP motifs in Pdx1ΔC embryos should eliminate SPOP-mediated ubiquitylation and promote protein stabilization, yet protein levels are decreased. This is likely explained by a concurrent reduction in *Pdx1* mRNA expression (Oliver-Krasinski et al., 2009), suggesting regulatory mechanisms beyond SPOP-mediated protein regulation. By contrast, the Pdx1Δ207 deletion leaves both SPOP motifs intact, preserving normal PDX1 protein turnover and levels. Although the Pdx1Δ216 deletion overlaps with SPOP motif 1, PDX1 protein levels remain normal, suggesting that partial deletion of the first motif may not abolish SPOP-mediated regulation; this warrants direct testing in future studies.

After determining that none of the PDX1 C-terminal constructs showed significant changes in PDX1 nuclear localization, we turned to CUT&RUN sequencing to assess whether deletion of residues 207-223 affects chromatin occupancy at PDX1 targets. These studies revealed a dramatic loss of chromatin occupancy in MIN6 β cells expressing Pdx1Δ207. Occupancy at approximately one-third of wild-type PDX1 sites was significantly lost and these were preferentially distributed at distal regulatory elements, co-occupied by other homeodomain factors such as NKX6.1 and FOXA2 in β cells (Ediger et al., 2017). Notably, although these studies were performed in the MIN6 β-cell line, genes associated with lost occupancy sites encompassed a broad program of developmental transcription factors crucial for pancreatic progenitor specification and endocrine development (*Foxa2*, *Isl1*, *Mnx1*, *Hnf1a* and *Hnf1b*) (Wang et al., 2018). This broader occupancy defect supports our *in vivo* findings that residues 207-223 are required across multiple stages of pancreatic development, not solely in differentiated β cells.

Our biochemical studies provide a direct molecular basis for the severity and multiphasic nature of the Pdx1Δ207 developmental phenotype. The chromatin occupancy comparison across the three PDX1 C-terminal mutants is particularly informative. Pdx1ΔC (truncated at residue 210) shows no loss of PDX1 occupancy, but impaired *Ins2* transactivation, whereas deletion of resides 207-223 results in dramatic occupancy loss and impaired transactivation. These findings localize the chromatin defect specifically to residues 207-210. In Pdx1Δ207 impaired transactivation is a consequence of lost chromatin occupancy, whereas in Pdx1ΔC transactivation is impaired despite retained occupancy, implicating C-terminal residues 211-284 as a separate domain required for transcriptional activation. This aligns with the known role of the PDX1 C-terminus in endocrine progenitor specification, where cooperativity with OC1 drives synergistic *Ngn3* activation and promotion of the endocrine gene landscape (Oliver-Krasinski et al., 2009). Whether Pdx1Δ207 retains its interaction with OC1 remains to be tested.

Previously published NMR analysis of human PDX1 (Bastidas and Showalter, 2013; Cook et al., 2019; Sahu et al., 2014), together with our disorder prediction tools (IUPRED3 and PrDOS) place the end of the conserved homeodomain at residue 206, and classify residues 207-210 as part of the PDX1 C-terminal IDR. Interestingly, AF3 predicts with high confidence that resides 207-210 are ordered, forming an extension of helix 3 within the PDX1 homeodomain. We hypothesize these discrepancies reflect methodological differences between the biophysical assays used or that they have uncovered novel aspects about the C-terminal IDR residues directly adjacent to the PDX1 homeodomain. Rather than full-length PDX1 protein, published NMR studies used protein fragments containing the canonical homeodomain (residues 146-206 of human PDX1, with the C-terminal end corresponding to residue 207 of mouse PDX1) or the C-terminus (residues 204-283 of human PDX1, which would include residues 205 and 206 of mouse PDX1). Our full-length predictions may therefore reveal the potential for a disorder-to-order structural switch at residues 207-210 that could be vital for PDX1 activity, similar to features previously observed in IDRs of other transcription factors, such as FoxM1 (Marceau et al., 2019). Indeed, it has been shown that the junction of ordered-disordered regions, such as the homeodomain-IDR junction at residues 207-210 we describe, serves as a crucial functional element of transcription factors (Brodsky et al., 2020). Future biophysical studies of full-length PDX1 or focused assessments fully inclusive of the homeodomain-IDR junction would be insightful to clarify the structural and functional features of these critical residues for PDX1 activity.

Notably, while AF3 predicts that residues 207-210 form an extended helix adjacent to the homeodomain, it does not predict direct DNA contacts. We propose several non-exclusive mechanisms for how residues 207-210 could influence chromatin binding independent of direct DNA contact. First, since PDX1 interacts with multiple co-factors, this region may mediate one or more of these interactions to promote chromatin accessibility and/or occupancy (Usher and Showalter, 2022). Second, although major-base specific contacts are retained, the predicted loss of the Lys201 backbone contact suggests this extension could instead stabilize PDX1 on DNA or modulate binding affinity. Furthermore, IDRs adjacent to DNA-binding domains can facilitate efficient searching of target loci, e.g. by sequestering co-factors for the formation of transcriptional hubs (Jonas et al., 2025).

The homeodomain-adjacent residues (DKKR) are unique to PDX1 among all homeobox subfamilies and are highly conserved across PDX1 orthologs of distantly related species of chordates. Although the remainder of the PDX1 C-terminus is largely poorly conserved, a small number of residues (mouse positions: 211 and 224-232), deleted in both Pdx1ΔC and Pdx1Δ216, are moderately conserved across chordate species, yet their loss has no impact on chromatin occupancy. This further supports the unique functional importance of residues 207-210 to PDX1 function.

Taken together, this study identifies a highly conserved region (207-210) that lies directly C-terminal to the canonical PDX1 homeodomain that is essential for optimal chromatin occupancy at direct PDX1 target loci and required for both pancreas organogenesis and β-cell development. Precise mapping of the functional boundaries within PDX1 is crucially important given the well-established role of *PDX1* variants in MODY4 and T2D susceptibility; the findings here provide a framework for interpretation of disease-associated mutations within key diabetes-susceptibility genes.

### Limitations of the study

Deletion of residues 207-223 removes a region with pronounced charge segregation (kappa: 0.331) and juxtaposes the homeodomain with the downstream C-terminal sequence, which lacks comparable charge segregation (kappa: 0.132) (Cook et al., 2019). This deletion also positions a proline immediately adjacent to the homeodomain; prolines are well known to act as helix breakers, introducing kinks or bends in adjacent secondary structure. Future studies will therefore need to determine whether the phenotypes observed in Pdx1Δ207 result from loss of the DKKR charge motif specifically or from disruption of the homeodomain helix integrity via the introduced proline, or a combination of both.

## MATERIALS AND METHODS

### Structural disorder predictions of Pdx1 protein

Mean disorder score was calculated from two independent disorder-prediction algorithms: IUPRED3 and PrDOS (Erdős et al., 2021; Ishida and Kinoshita, 2007). The long-range method of IUPRED3 was used, with no smoothing of the results. PrDOS was used with a standard 5% false positive rate. Protein sequences were obtained from UniProt IDs P52946 (mouse) and P52945 (human).

### Pdx1 mutant mouse models

Generation of the Pdx1ΔC genetically modified mouse line has been previously described (Oliver-Krasinski et al., 2009). Frozen sperm from Pdx1ΔC heterozygous male mice were obtained from the Jackson Laboratory and the line was re-derived at the Vanderbilt Genome Editing Resource (VGER) using *in vitro* fertilization (IVF) with C57BL/6J (stock 664) egg donors from the Jackson Laboratory. Pdx1ΔC mice were on a mixed SV129/C57BL/6J background. Both the Pdx1Δ207-223 and Pdx1Δ216-232 genetically modified mouse lines were generated using CRISPR-Cas9 genome editing. Two guide RNAs were generated by Synethgo, sgRNA 1 ‘tagtagcgggaccccgagtg’ and sgRNA 2 ‘ttgtgcggtgacctcgggcg’ and injected with Cas9 protein (Sigma) into 150-300 zygotes at the University of Michigan Molecular Genetics Core. Presence of the Pdx1 deletions was confirmed by in-house genotyping PCR (Table S4). Pdx1Δ207-223 and Pdx1Δ216-232 mice were backcrossed one generation onto a C57BL/6J background. The strain background of each of the three PDX1 mouse lines was confirmed by the Transnetyx MiniMUGA background analysis (Cordova, TN). All mice and embryos were genotyped by Transnetyx (Cordova, TN) using duplicate sample processing (real-time TaqMan PCR). Mice were maintained on a 12:12 h light-dark cycle and were provided food and water *ad libitum*. All mouse experiments were approved by the Institutional Animal Care and Use Committee (IACUC) of Vanderbilt University Medical Center (VUMC).

### Embryo handling and pancreas dissection

Beginning at 8 weeks age, heterozygous (+/Δ) males and females were set up late in the afternoon. The morning of the vaginal plug was defined as E0.5. The posterior foregut organs, whole embryo or pancreas alone were dissected in ice-cold phosphate-buffered saline (PBS) at their respective embryonic timepoints. To account for variability in litters, all results described in this study are of embryos from at least three different litters. Bright-field images of the posterior foregut organs were taken on a Nikon AZ 100M microscope using transmitted white light and the 2× Plan Flour/0.2 NA air objective lens. At E18.5, individual pancreata were dissected and weighed using a benchtop scale and reported in milligrams (mg).

### Protein extraction and western blotting

Individual pancreata from E18.5 embryos were flash frozen in liquid nitrogen and stored at −80°C. Upon thawing, individual pancreata were homogenized in Pierce RIPA Buffer supplemented with protease and phosphatase inhibitor cocktails using a Pellet Pestle Cordless Motor (Table S3). The total protein concentration of each sample was measured using the Pierce BCA Protein Assay Kit. Samples (30 μg) were heat shocked at 95°C before being resolved on a NuPage 4-12% Bis-Tris gel and transferred to nitrocellulose membranes using an iBlot2 at 20 V for 7 min (Table S3). Membranes were blocked in 5% non-fat milk for 1 h at room temperature followed by incubation with goat anti-Pdx1 that recognizes the N terminus (1:5000) overnight at 4°C, and HRP-conjugated anti-goat secondary (1:7500) for 1 h at room temperature. Membranes were then stripped for 20 min at room temperature using the Restore Western Blot Stripping Buffer and probed for the housekeeping gene anti-mouse β-actin (1:1000). Protein concentration was detected using the SuperSignal West Pico Plus Chemiluminescent Substrate and Bio-Rad ChemiDoc MP. Densitometric quantification of Pdx1 and protein was performed using FIJI (Table S3).

### Formalin-fixed paraffin-embedded immunofluorescence

Posterior foregut organs or whole embryos were fixed overnight at 4°C in 4% paraformaldehyde (PFA) washed in PBS and then stored in 70% ethanol at 4°C until processing. For processing, tissues were dehydrated in an ethanol series, cleared in xylenes and embedded in paraffin wax. The entire paraffin wax-embedded tissues were cut at 5 μm, placed on charged slides and heated on a slide warner at 37°C overnight. For E11.5, every other section containing pancreatic bud tissue was selected, for all other timepoints, three sections at least 90 μm (E15.5) or 150 μm (E18.5) apart were selected for each analysis. Hematoxylin and Eosin staining was performed as described in a previous study (Riley et al., 2015). Sections were deparaffinized in xylenes and rehydrated in an ethanol series. All tissue sections required antigen retrieval in sodium citrate buffer (pH 6.0) or Tris-EGTA Buffer (TEG pH 9.0) in the microwave or pressure cooker. Sections were blocked using 5% normal donkey serum (NDS) for 1 h at room temperature, incubated overnight at 4°C in primary antibody solution with the appropriate dilution (Table S2). Sections were washed, incubated with fluorescently conjugated species-specific secondary antibodies with the appropriate dilution (Table S2) for 2 h at room temperature and subsequently stained with 4′,6-diamidino-2-phenylindole (DAPI) (5 mg/ml) to label nuclei. Sections were washed in ddH_2_0 and mounted with 22×40 mm No.1 thickness coverslips and VECTASHIELD Vibrance Antifade Mounting Medium. Images were captured on a Nikon STORM microscope using conventional widefield fluorescence with a 20× Plan Apo 0.8 NA WD 1 mm objective lens. For every parameter, all cells on the entire section were counted manually using FIJI.

### Whole-mount immunofluorescence

Whole embryos (E11.5) or posterior foreguts (E15.5, E18.5) were fixed overnight at 4°C in 4% PFA on a rotator and washed in PBS. Individual foregut organs (E11.5) or pancreata (E15.5/E18.5) were permeabilized for 2 h a room temperature, blocked in 5% NDS for 1 h at room temperature and incubated with primary antibodies overnight (4°C) at the appropriate dilution (Table S2). The following day, samples were washed and incubated overnight with fluorescently conjugated secondaries (1:500) (4°C). Samples were again washed (4°C), dehydrated in methanol series and cleared in BABB (1:2 parts benzyl alcohol, benzyl benzoate) on a 35 mm glass bottom dish. Samples were visualized using a Nikon Yokogawa CSU-W1 spinning disk confocal microscope using a Plan Apochromat Lambda D, 20×, OFN25, DIC2, N.A. 0.80, W.D. 0.80 mm objective lens for E11.5 or Plan Apochromat Lambda D, 10×, OFN25, DIC2, N.A. 0.45, W.D. 4.0 mm objective lens for E15.5 and E18.5 with optical sections taken every 0.8 μm for E11.5 samples, every 2.5 μm for E15.5 pancreata, and every 6 μm for E18.5 pancreata.

### Quantification of pancreas bud and lumen volume

At E11.5, ventral and dorsal bud volumes were quantified based on PDX1 and ECAD co-localization within *z*-stacks using the Imaris 10.2 3D rendering software. The epithelial lumen volume was quantified for each bud based on MUC1 expression. The surface-rendering function on Imaris was used with ‘detail’ set to 2 μm in conjunction with the ‘smooth’ function to isolate each pancreatic bud.

### Quantification of plexus complexity and branching

The complexity and branching of the ductal plexus/network were analyzed for all pancreatic wholemounts using FIJI (Darrigrand et al., 2024). The MUC1 channel was isolated and a Gaussian blur was applied to the *z*-stack to smooth the images and to make the lumen network continuous. The Otsu thresholding method was used to adequately cover MUC1 expression throughout the entire *z*-stack. The skeletonize function was used to create the MUC1^+^ ductal skeleton. The analyze skeleton 2D/3D function was used to quantify the total number of branches, branch endpoints and plexus junctions.

### PDX1 C-terminal plasmid constructs

The N-terminus of each Pdx1 C-terminal mutant (Δ207, Δ216 and ΔC) was tagged with mCherry and cloned into the previously modified pSKII vector. In this vector, mCherry-Pdx1 expression was driven by the *Hsp* minimal promoter and the 1 kb PB islet enhancer from the *Pdx1* upstream regulatory sequences. The sequence and plasmid map for each PB-mCherry construct can be accessed at Addgene: mCherry wild-type PDX1, Addgene plasmid #261847; mCherry Pdx1∆207-223, Addgene plasmid #261848; mCherry Pdx1∆C, Addgene plasmid #261849; mCherry Pdx1∆216-232, Addgene plasmid #261850; mCherry-EV, Addgene plasmid #261851.

### Cell culture and transfection

MIN6 mouse insulinoma β-cell line were obtained prior to experiments from Sigma-Aldrich (SCC623), where they were authenticated and tested for contamination. Cells were cultured in Dulbecco's Modified Eagle's Medium (DMEM) supplemented with high glucose (4.5 g/l), 10% fetal bovine serum (FBS), L-glutamine and 1% penicillin-streptomycin (P/S). MIN6 cell passages (P) (P3-9) were transiently transfected with each mCherry-Pdx1 construct using a 1:2.5 (w/v) ratio of DNA (2.5 μg) to Lipofectamine 2000 (6.25 μls; ThermoFisher Scientific), diluted in Opti-MEM reduced serum medium. For the insulin promoter reporter activity, HEK293T cells (5×10^5^ cell per well in a 6-well plate), cultured in DMEM, were transfected with proximal insulin promoter vector (pGL410-Ins2500; 500 ng DNA) (Addgene #49058) (Kuroda et al., 2009) and transfection control beta-galactosidase (CMV-beta gal; 100 ng DNA) (a gift from Mitchell Lazar, University of Pennsylvania, Philadelphia, USA), and with different mCherry-PB-Pdx1 constructs (PDX1, Pdx1Δ207, Pdx1Δ216 and Pdx1ΔC) in an increasing dose (25, 125, 250, 500 ng DNA) using a 1:5 ratio of DNA to Lipofectamine 2000 (ThermoFisher Scientific), diluted in Opti-MEM reduced serum medium.

### Subcellular fractionation

48 h post-transfection, cells were treated with 0.05% Trypsin-EDTA and harvested for subcellular fractionation using the NE-PER Nuclear and Cytoplasmic Extraction Reagents. Samples (2 μg) were resolved on a NuPage 4%-12% Bis-Tris gel and transferred to nitrocellulose membranes. Membranes incubated with the following antibodies DsRed (mCherry), PARP (nuclear fraction) and GAPDH (cytoplasmic fraction) (Table S2).

### Live-cell imaging

MIN6 cells were grown on 35 mm glass bottom dishes with #1.5 polymer coverslip coated with poly-d-lysine (PDL) for 24 h prior to transfection to promote adhesion and attachment. 48 h post-transfection, cells were co-labeled with 1 μg/ml Hoechst (33342) for 20 min at 37°C. The cells were imaged on the Nikon Yokogawa CSU-W1 spinning disk confocal microscope using a Plan Apochromat Lambda D 60× Oil Immersion Objective Lens, N.A. 1.42, W.D. 0.15 mm, DIC.

### CUT&RUN sequencing

CUT&RUN was performed on 500,000 dispersed MIN6 cells per condition using the CUTANA ChIC/CUT&RUN Kit v6 (Table S3). After cells were immobilized on Concanavalin-A beads, samples were incubated with 0.5 μg of rabbit anti-DsRed or rabbit anti-H3K4me3 antibodies overnight at 4°C. pAG-MNase was added to cells at room temperature for 10 min and targeted chromatin digestion was induced by adding 100 mM CaCl_2_ for 2 h at 4°C. Following chromatin digestion, 0.5 μg of *E. coli* Spike-in DNA was added to each sample for improved CUT&RUN experimental normalization (Meers et al., 2019). DNA fragments were purified using the CUTANA ChIC/CUT&RUN Kit v6. DNA was resuspended in 0.1×TE Buffer and used for library preparation with the NEBNext Ultra II DNA Library Kit for Illumina. Index matching was performed using NEBNext Unique Dual Index Primer Pairs (Table S3). Libraries were sequenced as PE150 reads on the Novaseq X platform at the Vanderbilt University Medical Center Vanderbilt Technologies for Advanced Genomics(VANTAGE) Core.

### CUT&RUN-seq processing and analysis

All libraries had >28 million reads and were processed using the following bioinformatics workflow. Raw reads were adapter trimmed using Trimmomatic v0.39 with TruSeq CD adapter sequences. Trimmed paired-end reads were aligned simultaneously to the combined mm39 (GRCm39) mouse genome and *E. coli* K-12 (MG1655) reference genome using Bowtie2 v2.5.4 with default parameters. Aligned reads were converted to BAM format using SAMtools v1.22.1, filtered to removed unmapped reads and low-quality alignments, sorted and indexed. Mouse mapping reads were extracted by retaining only canonical chromosomes. E. coli-mapping reads were counted for each sample and used as spike-in controls for normalization. Peak calling was performed using MACS3 v3.0.3 with paired-end BAM format and mouse genome size. Peaks were filtered against the mm39 ENCODE blacklist regions using bedtools intersect v2.31.1. Condition-specific consensus peak sets were generated requiring a minimum overlap of two replicates using DiffBind v3.20.0. Read counts over consensus peaks were quantified using DiffBind with paired-end read counting. Genome-wide differential binding analysis was performed using DESeq2 v1.50.2 with spike-in normalization scale factors, with factors calculated as the ratio of the within-condition mean *E. coli* read counts to the individual sample *E. coli* read counts, replacing the default median-of-ratios normalization method. Significant differential peaks were defined as adjusted p-value <0.05 and | log2 fold change | ≥0.585. Peak annotation was performed using ChIPseeker v1.46.1 with the UCSC mm39 RefGene annotation database. *De novo* and known motif analysis was performed using HOMER v5.1 with the mm39 genome against 487 known transcription factor motifs (Heinz et al., 2010). All analyses were performed in R v4.5.3. Merged spike-in normalized bigwig files were generated using deeptools bamCoverage v3.5.6 with the spike-in scale factors as described above. Sequencing tracks were visualized using GRCm39/mm39 mouse genome on the integrative genomics viewer (IGV) v2.19.7 with the spike-in normalized bigwig files for all Pdx1 mutant conditions, H3K4me3 positive control and mCherry-EV negative control.

### Luciferase assay

48 h post-transfection, HEK293T cells were washed with ice-cold PBS twice, and cells were lysed with 150 μl of 1× reporter lysis buffer (E4030; Promega). Cells were then scraped from each of the 6-well plates and transferred to the microcentrifuge tubes on ice. Microcentrifuge tubes were briefly vortexed, centrifuged at 12,000 ***g*** for 2 min (at 4°C), and supernatant was transferred to the new tubes to be stored at −80°C and/or used for the luciferase assay using The GloMax Discover System (Promega) plate reader following the pre-installed protocol “Luciferase Assay System”. For the assessment of beta-galactosidase levels, in 96-well plates, 2 μl of cell lysates and 150 μl of 2.17 mM ONPG (2-Nitrophenyl-beta-D-galactosidase) (ThermoFisher Scientific) in Z buffer (60 mM Na_2_HPO_4_, 40 mM NaHPO_4_, 10 mM KCl) were incubated for 10 min at room temperature and for 5 min at 37°C. The absorbance, at 450 nm, was measured using The GloMax Discover System (Promega), and, after background correction, the data were normalized to beta-galactosidase.

### AlphaFold3 prediction

The protein sequence for mouse Pdx1 (P52946) was downloaded from UniProt and uploaded to the AlphaFold3 (AF3) server (https://alphafoldserver.com/) for structure prediction. Predictions were performed for wild-type Pdx1 as well as three C-terminal truncation mutants (Δ207, Δ216 and ΔC) using default server parameters. The resulting models were visualized and analyzed using UCSF ChimeraX (Meng et al., 2023). Models of wild-type PDX1 and each PDX1 C-terminal deletion mutant in complex with the 22 bp DNA duplex containing the *Ins2 A1* (sequence: GCCCTTAATGGGTCAAACAGCA) were generated using AlphaFold3 (*n*=2500), 500 seeds; 5 models per seed. Structures were visualized and analyzed in ChimeraX. Protein-DNA contacts were identified using the ‘contacts’ command (restrict to protein/DNA chains, default van der Waals overlap cutoff) to confirm overall interface engagement. To distinguish base-specific from backbone mediated contacts, residue-level contact analysis was performed by selecting DNA base atoms (N1, N3, N7, N9, C2, C4-6, C8, O2, O4, O6, N2, N4 and N6) or backbone/phosphate atoms (P, OP1, OP2 and sugar-phosphate ring atoms) and identifying protein chain A atoms within 4.0 Å using the ‘zone’ command. Contacting residues were charted for each of the 2500 AF3 models per PDX1 mutant, and reported as the percentage of models showing contact within 4.0 Å.

### Multiple sequence alignments

Mouse PDX1 protein sequence and homeodomain-containing transcription factors were retrieved from Uniprot databases (Table S1). Orthologous sequences from multiple species, including vertebrates (*Anolis carolinensis, Chrysemys picta belli, Danio rerio, Gallus gallus, Homo sapiens*, *Mus musculus* and *Xenopus laevis*) and an invertebrate (*Branchiostoma lanceolatum*), were collected for comparative analysis (Table S1). Multiple sequence alignments were performed using MAFFT with default parameters and visualized in Jalview v2.11.5.2. with percentage identity applied to the helix residues of the canonical homeodomain and Zappo coloring scheme applied to the DKKR motif to highlight physiochemical properties of the residues. A phylogenetic tree was constructed from the mouse PDX1 and HOX gene cluster alignment using neighbor joining with the BLOSUM62 substitution matrix. The tree was visualized using Jalview. Sequence conservation was assessed using the default alignment conservation metrics (JAAWS2.2/AACon1.1) in Jalview.

### Statistics

All data are presented as the mean±standard error of the mean (s.e.m.). All statistical analysis was performed in GraphPad Prism using a one-way ANOVA, with Tukey's post-hoc multiple comparisons test to compare each genotype.

## Supplementary Material

10.1242/develop.205842_sup1 Supplementary information

## References

[DEV205842C1] Abramson, J., Adler, J., Dunger, J., Evans, R., Green, T., Pritzel, A., Ronneberger, O., Willmore, L., Ballard, A. J., Bambrick, J. et al. (2024). Accurate structure prediction of biomolecular interactions with AlphaFold 3. *Nature* 630, 493-500. 10.1038/s41586-024-07487-w PMC11168924 38718835

[DEV205842C2] Ahlgren, U., Jonsson, J. and Edlund, H. (1996). The morphogenesis of the pancreatic mesenchyme is uncoupled from that of the pancreatic epithelium in IPF1/PDX1-deficient mice. *Development* 122, 1409-1416. 10.1242/dev.122.5.1409 8625829

[DEV205842C3] Artner, I., Hang, Y., Mazur, M., Yamamoto, T., Guo, M., Lindner, J., Magnuson, M. A. and Stein, R. (2010). MafA and MafB regulate genes critical to beta-cells in a unique temporal manner. *Diabetes* 59, 2530-2539. 10.2337/db10-0190 PMC3279542 20627934

[DEV205842C4] Banerjee-Basu, S. and Baxevanis, A. D. (2001). Molecular evolution of the homeodomain family of transcription factors. *Nucleic Acids Res.* 29, 3258-3269. 10.1093/nar/29.15.3258 PMC55828 11470884

[DEV205842C5] Bastidas, M. and Showalter, S. A. (2013). Thermodynamic and structural determinants of differential Pdx1 binding to elements from the insulin and IAPP promoters. *J. Mol. Biol.* 425, 3360-3377. 10.1016/j.jmb.2013.06.011 PMC3800017 23796517

[DEV205842C6] Brissova, M., Shiota, M., Nicholson, W. E., Gannon, M., Knobel, S. M., Piston, D. W., Wright, C. V. E. and Powers, A. C. (2002). Reduction in pancreatic transcription factor PDX-1 impairs glucose-stimulated insulin secretion. *J. Biol. Chem.* 277, 11225-11232. 10.1074/jbc.M111272200 11781323

[DEV205842C7] Brodsky, S., Jana, T., Mittelman, K., Chapal, M., Kumar, D. K., Carmi, M. and Barkai, N. (2020). Intrinsically disordered regions direct transcription factor in vivo binding specificity. *Mol. Cell* 79, 459-471.e454. 10.1016/j.molcel.2020.05.032 32553192

[DEV205842C8] Claiborn, K. C., Sachdeva, M. M., Cannon, C. E., Groff, D. N., Singer, J. D. and Stoffers, D. A. (2010). Pcif1 modulates Pdx1 protein stability and pancreatic β cell function and survival in mice. *J. Clin. Invest.* 120, 3713-3721. 10.1172/JCI40440 PMC2947215 20811152

[DEV205842C9] Cockburn, B. N., Bermano, G., Boodram, L.-L. G., Teelucksingh, S., Tsuchiya, T., Mahabir, D., Allan, A. B., Stein, R., Docherty, K. and Bell, G. I. (2004). Insulin promoter factor-1 mutations and diabetes in Trinidad: identification of a novel diabetes-associated mutation (E224K) in an Indo-Trinidadian family. *J. Clin. Endocrinol. Metab.* 89, 971-978. 10.1210/jc.2003-031282 14764823

[DEV205842C10] Cook, E. C., Sahu, D., Bastidas, M. and Showalter, S. A. (2019). Solution ensemble of the C-terminal domain from the transcription factor Pdx1 resembles an excluded volume polymer. *J. Phys. Chem. B* 123, 106-116. 10.1021/acs.jpcb.8b10051 PMC7874520 30525611

[DEV205842C11] Cyphert, H. A., Walker, E. M., Hang, Y., Dhawan, S., Haliyur, R., Bonatakis, L., Avrahami, D., Brissova, M., Kaestner, K. H., Bhushan, A. et al. (2019). Examining how the MAFB transcription factor affects islet β-cell function postnatally. *Diabetes* 68, 337-348. 10.2337/db18-0903 PMC6341297 30425060

[DEV205842C12] Darrigrand, J. F., Salowka, A., Torres-Cano, A., Tapia-Rojo, R., Zhu, T., Garcia-Manyes, S. and Spagnoli, F. M. (2024). Acinar-ductal cell rearrangement drives branching morphogenesis of the murine pancreas in an IGF/PI3K-dependent manner. *Dev. Cell* 59, 326-338.e325. 10.1016/j.devcel.2023.12.011 PMC11805742 38237591

[DEV205842C13] Ediger, B. N., Lim, H.-W., Juliana, C., Groff, D. N., Williams, L. T., Dominguez, G., Liu, J.-H., Taylor, B. L., Walp, E. R., Kameswaran, V. et al. (2017). LIM domain-binding 1 maintains the terminally differentiated state of pancreatic β cells. *J. Clin. Invest.* 127, 215-229. 10.1172/JCI88016 PMC5199701 27941246

[DEV205842C14] Erdős, G., Pajkos, M. and Dosztányi, Z. (2021). IUPred3: prediction of protein disorder enhanced with unambiguous experimental annotation and visualization of evolutionary conservation. *Nucleic Acids Res.* 49, W297-W303. 10.1093/nar/gkab408 PMC8262696 34048569

[DEV205842C16] Gannon, M., Ables, E. T., Crawford, L., Lowe, D., Offield, M. F., Magnuson, M. A. and Wright, C. V. (2008). pdx-1 function is specifically required in embryonic beta cells to generate appropriate numbers of endocrine cell types and maintain glucose homeostasis. *Dev. Biol.* 314, 406-417. 10.1016/j.ydbio.2007.10.038 PMC2269701 18155690

[DEV205842C17] Gao, T., McKenna, B., Li, C., Reichert, M., Nguyen, J., Singh, T., Yang, C., Pannikar, A., Doliba, N., Zhang, T. et al. (2014). Pdx1 maintains β cell identity and function by repressing an α cell program. *Cell Metab.* 19, 259-271. 10.1016/j.cmet.2013.12.002 PMC3950964 24506867

[DEV205842C18] Gragnoli, C., Stanojevic, V., Gorini, A., Von Preussenthal, G. M., Thomas, M. K. and Habener, J. F. (2005). IPF-1/MODY4 gene missense mutation in an Italian family with type 2 and gestational diabetes. *Metabolism* 54, 983-988. 10.1016/j.metabol.2005.01.037 16092045

[DEV205842C19] Gregg, B. E., Moore, P. C., Demozay, D., Hall, B. A., Li, M., Husain, A., Wright, A. J., Atkinson, M. A. and Rhodes, C. J. (2012). Formation of a human β-cell population within pancreatic islets is set early in life. *J. Clin. Endocrinol. Metab.* 97, 3197-3206. 10.1210/jc.2012-1206 PMC3431572 22745242

[DEV205842C20] Gu, G., Brown, J. R. and Melton, D. A. (2003). Direct lineage tracing reveals the ontogeny of pancreatic cell fates during mouse embryogenesis. *Mech. Dev.* 120, 35-43. 10.1016/S0925-4773(02)00330-1 12490294

[DEV205842C21] Hani, E. H., Stoffers, D. A., Chèvre, J. C., Durand, E., Stanojevic, V., Dina, C., Habener, J. F. and Froguel, P. (1999). Defective mutations in the insulin promoter factor-1 (IPF-1) gene in late-onset type 2 diabetes mellitus. *J. Clin. Invest.* 104, R41-R48. 10.1172/JCI7469 PMC409821 10545531

[DEV205842C22] Heinz, S., Benner, C., Spann, N., Bertolino, E., Lin, Y. C., Laslo, P., Cheng, J. X., Murre, C., Singh, H. and Glass, C. K. (2010). Simple combinations of lineage-determining transcription factors prime cis-regulatory elements required for macrophage and B cell identities. *Mol. Cell* 38, 576-589. 10.1016/j.molcel.2010.05.004 PMC2898526 20513432

[DEV205842C23] Henley, K. D., Stanescu, D. E., Kropp, P. A., Wright, C. V. E., Won, K. J., Stoffers, D. A. and Gannon, M. (2016). Threshold-dependent cooperativity of Pdx1 and Oc1 in pancreatic progenitors establishes competency for endocrine differentiation and β-cell function. *Cell Rep.* 15, 2637-2650. 10.1016/j.celrep.2016.05.040 PMC4917419 27292642

[DEV205842C24] Hogan, B. L. (1999). Morphogenesis. *Cell* 96, 225-233. 10.1016/S0092-8674(00)80562-0 9988217

[DEV205842C25] Ishida, T. and Kinoshita, K. (2007). PrDOS: prediction of disordered protein regions from amino acid sequence. *Nucleic Acids Res.* 35, W460-W464. 10.1093/nar/gkm363 PMC1933209 17567614

[DEV205842C26] Jonas, F., Navon, Y. and Barkai, N. (2025). Intrinsically disordered regions as facilitators of the transcription factor target search. *Nat. Rev. Genet.* 26, 424-435. 10.1038/s41576-025-00816-3 39984675

[DEV205842C27] Jonsson, J., Carlsson, L., Edlund, T. and Edlund, H. (1994). Insulin-promoter-factor 1 is required for pancreas development in mice. *Nature* 371, 606-609. 10.1038/371606a0 7935793

[DEV205842C28] Kropp, P. A., Dunn, J. C., Carboneau, B. A., Stoffers, D. A. and Gannon, M. (2018). Cooperative function of Pdx1 and Oc1 in multipotent pancreatic progenitors impacts postnatal islet maturation and adaptability. *Am. J. Physiol. Endocrinol. Metab.* 314, E308-E321. 10.1152/ajpendo.00260.2017 PMC5966755 29351489

[DEV205842C29] Kuroda, A., Rauch, T. A., Todorov, I., Ku, H. T., Al-Abdullah, I. H., Kandeel, F., Mullen, Y., Pfeifer, G. P. and Ferreri, K. (2009). Insulin gene expression is regulated by DNA methylation. *PLoS ONE* 4, e6953. 10.1371/journal.pone.0006953 PMC2735004 19742322

[DEV205842C30] Linnemann, A. K., Baan, M. and Davis, D. B. (2014). Pancreatic β-cell proliferation in obesity. *Adv. Nutr.* 5, 278-288. 10.3945/an.113.005488 PMC4013180 24829474

[DEV205842C31] Liu, A., Oliver-Krasinski, J. and Stoffers, D. A. (2006). Two conserved domains in PCIF1 mediate interaction with pancreatic transcription factor PDX-1. *FEBS Lett.* 580, 6701-6706. 10.1016/j.febslet.2006.11.021 17126328

[DEV205842C32] Longo, A., Guanga, G. P. and Rose, R. B. (2007). Structural basis for induced fit mechanisms in DNA recognition by the Pdx1 homeodomain. *Biochemistry* 46, 2948-2957. 10.1021/bi060969l 17315980

[DEV205842C33] Love, M. I., Huber, W. and Anders, S. (2014). Moderated estimation of fold change and dispersion for RNA-seq data with DESeq2. *Genome Biol.* 15, 550. 10.1186/s13059-014-0550-8 PMC4302049 25516281

[DEV205842C34] Marceau, A. H., Brison, C. M., Nerli, S., Arsenault, H. E., McShan, A. C., Chen, E., Lee, H.-W., Benanti, J. A., Sgourakis, N. G. and Rubin, S. M. (2019). An order-to-disorder structural switch activates the FoxM1 transcription factor. *eLife* 8, e46131. 10.7554/eLife.46131 PMC6538375 31134895

[DEV205842C35] Marty-Santos, L. and Cleaver, O. (2016). Pdx1 regulates pancreas tubulogenesis and E-cadherin expression. *Development* 143, 1056. 10.1242/dev.135806 PMC4813289 26980795

[DEV205842C36] Meers, M. P., Bryson, T. D., Henikoff, J. G. and Henikoff, S. (2019). Improved CUT&RUN chromatin profiling tools. *eLife* 8, e46314. 10.7554/eLife.46314 PMC6598765 31232687

[DEV205842C37] Meng, E. C., Goddard, T. D., Pettersen, E. F., Couch, G. S., Pearson, Z. J., Morris, J. H. and Ferrin, T. E. (2023). UCSF ChimeraX: tools for structure building and analysis. *Protein Sci.* 32, e4792. 10.1002/pro.4792 PMC10588335 37774136

[DEV205842C38] Moede, T., Leibiger, B., Pour, H. G., Berggren, P. and Leibiger, I. B. (1999). Identification of a nuclear localization signal, RRMKWKK, in the homeodomain transcription factor PDX-1. *FEBS Lett.* 461, 229-234. 10.1016/S0014-5793(99)01446-5 10567702

[DEV205842C39] Offield, M. F., Jetton, T. L., Labosky, P. A., Ray, M., Stein, R. W., Magnuson, M. A., Hogan, B. L. M. and Wright, C. V. E. (1996). PDX-1 is required for pancreatic outgrowth and differentiation of the rostral duodenum. *Development* 122, 983-995. 10.1242/dev.122.3.983 8631275

[DEV205842C40] Oliver-Krasinski, J. M., Kasner, M. T., Yang, J., Crutchlow, M. F., Rustgi, A. K., Kaestner, K. H. and Stoffers, D. A. (2009). The diabetes gene Pdx1 regulates the transcriptional network of pancreatic endocrine progenitor cells in mice. *J. Clin. Invest.* 119, 1888-1898. 10.1172/JCI37028 PMC2701861 19487809

[DEV205842C41] Pictet, R. L., Clark, W. R., Williams, R. H. and Rutter, W. J. (1972). An ultrastructural analysis of the developing embryonic pancreas. *Dev. Biol.* 29, 436-467. 10.1016/0012-1606(72)90083-8 4570759

[DEV205842C42] Puri, S. and Hebrok, M. (2007). Dynamics of embryonic pancreas development using real-time imaging. *Dev. Biol.* 306, 82-93. 10.1016/j.ydbio.2007.03.003 PMC1988845 17448459

[DEV205842C43] Qiu, W. L., Zhang, Y. W., Feng, Y., Li, L. C., Yang, L. and Xu, C. R. (2017). Deciphering pancreatic islet β cell and α cell maturation pathways and characteristic features at the single-cell level. *Cell Metab.* 25, 1194-1205.e1194. 10.1016/j.cmet.2017.04.003 28467935

[DEV205842C44] Ramírez, F., Ryan, D. P., Grüning, B., Bhardwaj, V., Kilpert, F., Richter, A. S., Heyne, S., Dündar, F. and Manke, T. (2016). deepTools2: a next generation web server for deep-sequencing data analysis. *Nucleic Acids Res.* 44, W160-W165. 10.1093/nar/gkw257 PMC4987876 27079975

[DEV205842C45] Riley, K. G., Pasek, R. C., Maulis, M. F., Dunn, J. C., Bolus, W. R., Kendall, P. L., Hasty, A. H. and Gannon, M. (2015). Macrophages are essential for CTGF-mediated adult β-cell proliferation after injury. *Mol. Metab.* 4, 584-591. 10.1016/j.molmet.2015.05.002 PMC4529497 26266091

[DEV205842C46] Roy, N., Takeuchi, K. K., Ruggeri, J. M., Bailey, P., Chang, D., Li, J., Leonhardt, L., Puri, S., Hoffman, M. T., Gao, S. et al. (2016). PDX1 dynamically regulates pancreatic ductal adenocarcinoma initiation and maintenance. *Genes Dev.* 30, 2669-2683. 10.1101/gad.291021.116 PMC5238727 28087712

[DEV205842C47] Sahu, D., Bastidas, M. and Showalter, S. A. (2014). Generating NMR chemical shift assignments of intrinsically disordered proteins using carbon-detected NMR methods. *Anal. Biochem.* 449, 17-25. 10.1016/j.ab.2013.12.005 PMC3944900 24333248

[DEV205842C48] Stanger, B. Z., Tanaka, A. J. and Melton, D. A. (2007). Organ size is limited by the number of embryonic progenitor cells in the pancreas but not the liver. *Nature* 445, 886-891. 10.1038/nature05537 17259975

[DEV205842C49] Stanojevic, V., Habener, J. F. and Thomas, M. K. (2004). Pancreas duodenum homeobox-1 transcriptional activation requires interactions with p300. *Endocrinology* 145, 2918-2928. 10.1210/en.2003-1188 15001545

[DEV205842C50] Steinthorsdottir, V., Thorleifsson, G., Sulem, P., Helgason, H., Grarup, N., Sigurdsson, A., Helgadottir, H. T., Johannsdottir, H., Magnusson, O. T., Gudjonsson, S. A. et al. (2014). Identification of low-frequency and rare sequence variants associated with elevated or reduced risk of type 2 diabetes. *Nat. Genet.* 46, 294-298. 10.1038/ng.2882 24464100

[DEV205842C51] Stoffers, D. A., Ferrer, J., Clarke, W. L. and Habener, J. F. (1997a). Early-onset type-II diabetes mellitus (MODY4) linked to IPF1. *Nat. Genet.* 17, 138-139. 10.1038/ng1097-138 9326926

[DEV205842C52] Stoffers, D. A., Zinkin, N. T., Stanojevic, V., Clarke, W. L. and Habener, J. F. (1997b). Pancreatic agenesis attributable to a single nucleotide deletion in the human IPF1 gene coding sequence. *Nat. Genet.* 15, 106-110. 10.1038/ng0197-106 8988180

[DEV205842C53] Swift, G. H., Liu, Y., Rose, S. D., Bischof, L. J., Steelman, S., Buchberg, A. M., Wright, C. V. E. and MacDonald, R. J. (1998). An endocrine-exocrine switch in the activity of the pancreatic homeodomain protein PDX1 through formation of a trimeric complex with PBX1b and MRG1 (MEIS2). *Mol. Cell. Biol.* 18, 5109-5120. 10.1128/MCB.18.9.5109 PMC109096 9710595

[DEV205842C54] Tompa, P. (2012). Intrinsically disordered proteins: a 10-year recap. *Trends Biochem. Sci.* 37, 509-516. 10.1016/j.tibs.2012.08.004 22989858

[DEV205842C55] Usher, E. T. and Showalter, S. A. (2022). Biophysical insights into glucose-dependent transcriptional regulation by PDX1. *J. Biol. Chem.* 298, 102623. 10.1016/j.jbc.2022.102623 PMC9691942 36272648

[DEV205842C56] Usher, E. T., Sabri, N., Rohac, R., Boal, A. K., Mittag, T. and Showalter, S. A. (2021). Intrinsically disordered substrates dictate SPOP subnuclear localization and ubiquitination activity. *J. Biol. Chem.* 296, 100693. 10.1016/j.jbc.2021.100693 PMC8138767 33894201

[DEV205842C57] Uversky, V. N. (2019). Intrinsically disordered proteins and their “mysterious” (meta)physics. *Front. Phys.* 7, 10. 10.3389/fphy.2019.00010

[DEV205842C58] Villasenor, A., Chong, D. C., Henkemeyer, M. and Cleaver, O. (2010). Epithelial dynamics of pancreatic branching morphogenesis. *Development* 137, 4295-4305. 10.1242/dev.052993 PMC2990215 21098570

[DEV205842C59] Wang, X., Sterr, M., Burtscher, I., Chen, S., Hieronimus, A., Machicao, F., Staiger, H., Häring, H.-U., Lederer, G., Meitinger, T. et al. (2018). Genome-wide analysis of PDX1 target genes in human pancreatic progenitors. *Mol. Metab.* 9, 57-68. 10.1016/j.molmet.2018.01.011 PMC5870105 29396371

[DEV205842C60] Weng, J., Macfarlane, W. M., Lehto, M., Gu, H. F., Shepherd, L. M., Ivarsson, S. A., Wibell, L., Smith, T. and Groop, L. C. (2001). Functional consequences of mutations in the MODY4 gene (IPF1) and coexistence with MODY3 mutations. *Diabetologia* 44, 249-258. 10.1007/s001250051608 11270685

[DEV205842C61] Wu, K. L., Gannon, M., Peshavaria, M., Offield, M. F., Henderson, E., Ray, M., Marks, A., Gamer, L. W., Wright, C. V. E. and Stein, R. (1997). Hepatocyte nuclear factor 3beta is involved in pancreatic beta-cell-specific transcription of the pdx-1 gene. *Mol. Cell. Biol.* 17, 6002-6013. 10.1128/MCB.17.10.6002 PMC232449 9315659

[DEV205842C62] Yu, G., Wang, L. G. and He, Q. Y. (2015). ChIPseeker: an R/Bioconductor package for ChIP peak annotation, comparison and visualization. *Bioinformatics* 31, 2382-2383. 10.1093/bioinformatics/btv145 25765347

[DEV205842C63] Zhang, H., Ables, E. T., Pope, C. F., Washington, M. K., Hipkens, S., Means, A. L., Path, G., Seufert, J., Costa, R. H., Leiter, A. B. et al. (2009). Multiple, temporal-specific roles for HNF6 in pancreatic endocrine and ductal differentiation. *Mech. Dev.* 126, 958-973. 10.1016/j.mod.2009.09.006 PMC2783291 19766716

[DEV205842C64] Zhu, X., Oguh, A., Gingerich, M. A., Soleimanpour, S. A., Stoffers, D. A. and Gannon, M. (2021). Cell cycle regulation of the Pdx1 transcription factor in developing pancreas and insulin-producing β-cells. *Diabetes* 70, 903-916. 10.2337/db20-0599 PMC7980191 33526589

