# Supplementary material for: Conserved C-terminal residues adjacent to the canonical PDX1 homeodomain are required for chromatin occupancy and pancreas development
Source: Development. 2026 Oct 20;153(20):dev205842. doi: 10.1242/dev.205842 (PMC13723409; doi:10.1242/dev.205842)
Supplement: Supplementary file 1 [file develop-153-205842-s1.pdf]

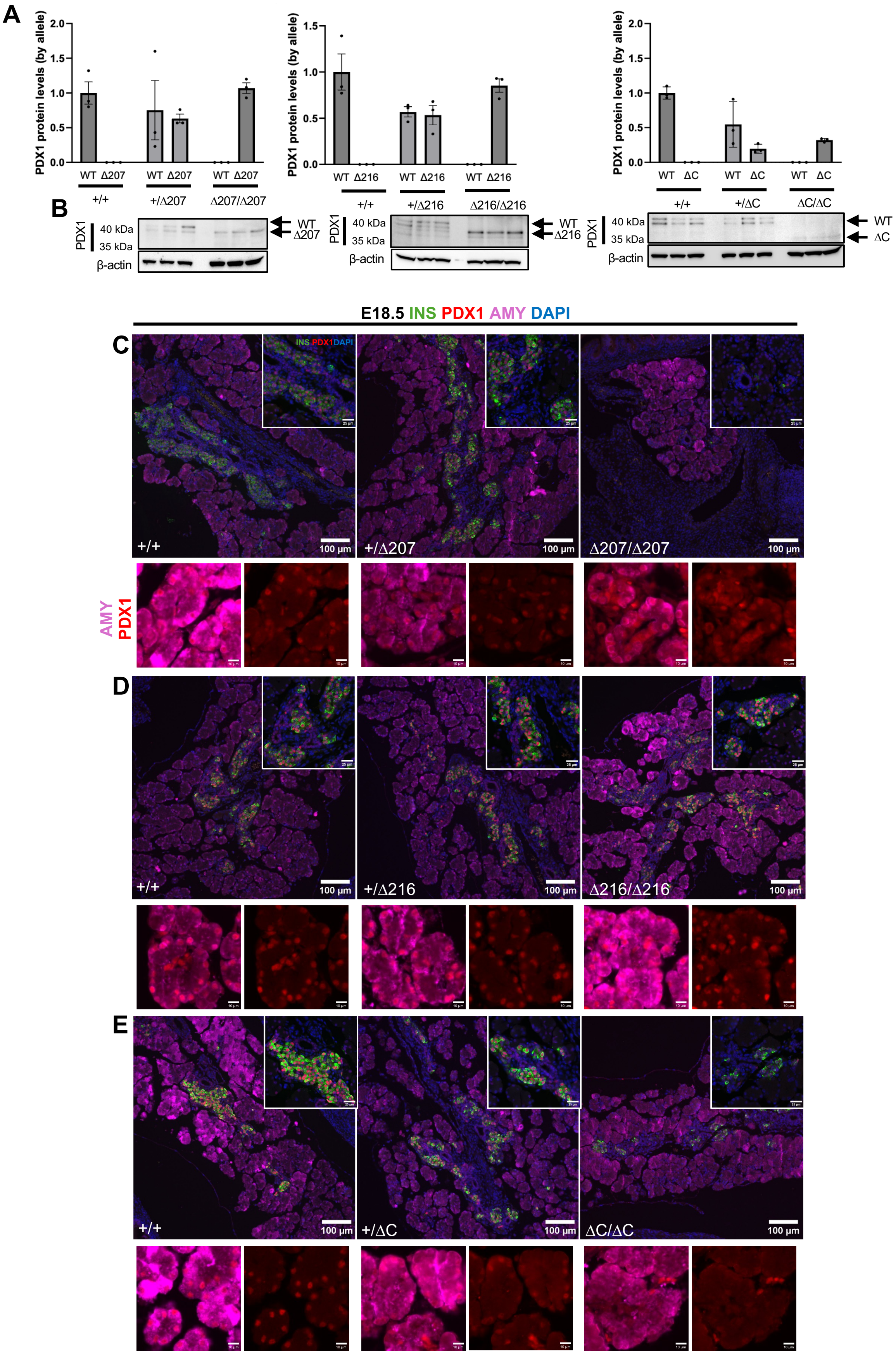

Fig. S1. E18.5 PDX1 protein levels within PDX1 C-terminal mutants

(A) Densitometric quantification of PDX1 protein at E18.5 for each allele relative to β-actin and normalized to control littermates (*n*=3). (B) Western blots of PDX1 and β-actin protein extracted from whole pancreas lysates at E18.5 for each PDX1 C-terminal mutant (*n*=3). (C-E) Representative images of E18.5 tissue-sections immunolabeled for insulin (green), PDX1 (red), AMY (magenta) and DAPI (blue) taken at 10x magnification. Inserts are taken at 20x magnification, where scale bar in insert displaying INS and PDX1 co-expression is 25 µm, and 10 µm in insert displaying AMY and Pdx1 co-expression.

Development • Supplementary information

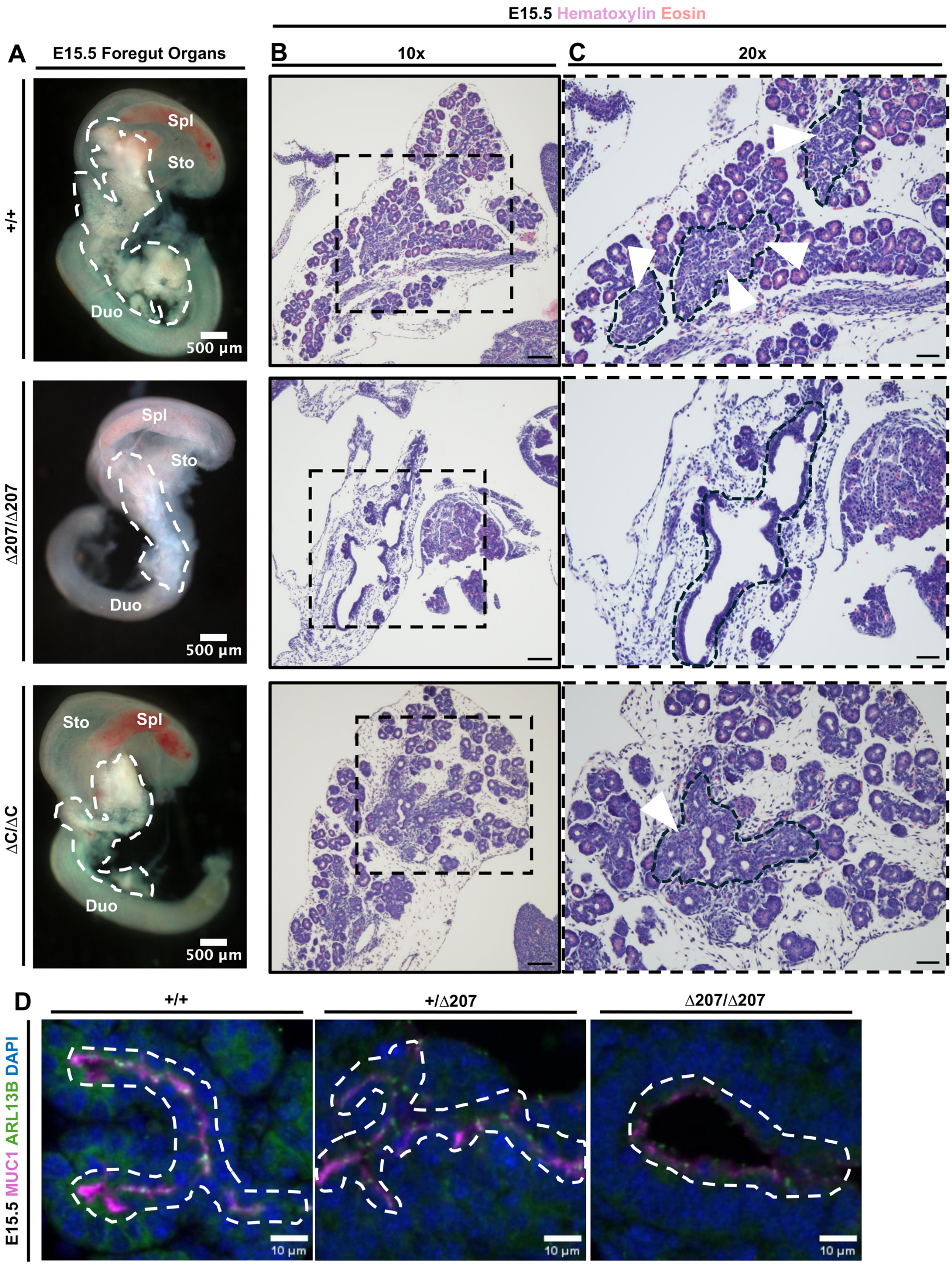

Fig. S2. Gross pancreas morphology of PDX1 C-terminal mutants at E15.5

(A) Representative images of E15.5 foregut organs. Pancreas tissue is outlined in white Sto: stomach, Spl: spleen, Duo: duodenum. (B)Tissue-sections from E15.5 pancreata stained with H&E. Representative brightfield images at 10x magnification showing overall pancreas size and gross morphology, scale bar is 50 µm. Boxed area is magnified in C. (C) 20x magnification displaying the ductal epithelium (outlined in black), scale bar is 50 µm. (D) Tissue-sections of E15.5 control and Pdx1Δ207 mutant pancreata immunolabeled for ARL13B (green), MUC1 (magenta) and DAPI (blue). Dashed white lines highlight ductal regions.

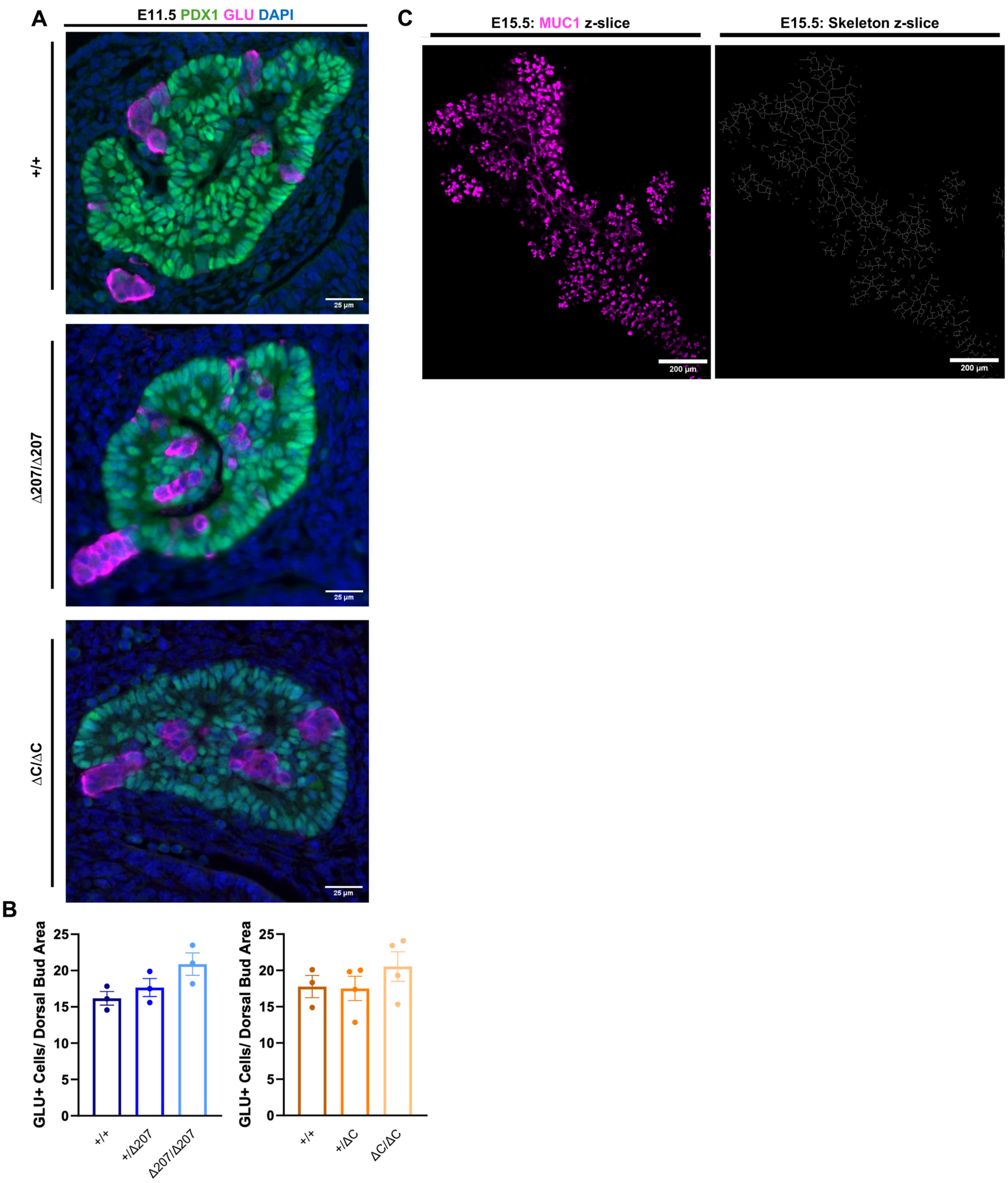

Fig. S3. The PDX1 C-terminal focal IDR is not required for the formation of primary wave endocrine cells.

(A) E11.5 tissue sections immunolabeled for PDX1 (green) and glucagon (GLU; magenta). (B) Quantification of the total number of GLU+ cells per dorsal bud area (µm^2 x 10^{-4}). Each dot represents a single embryo (n=3-4) (every other tissue section containing pancreas bud was quantified) (C) Representative FIJI z-slice of a control E15.5 MUC1 skeleton. Significant p-values are displayed; absence of a value indicates no significant difference (one-way ANOVA with Tukey's). Bars indicate mean±s.e.m.

Development • Supplementary information

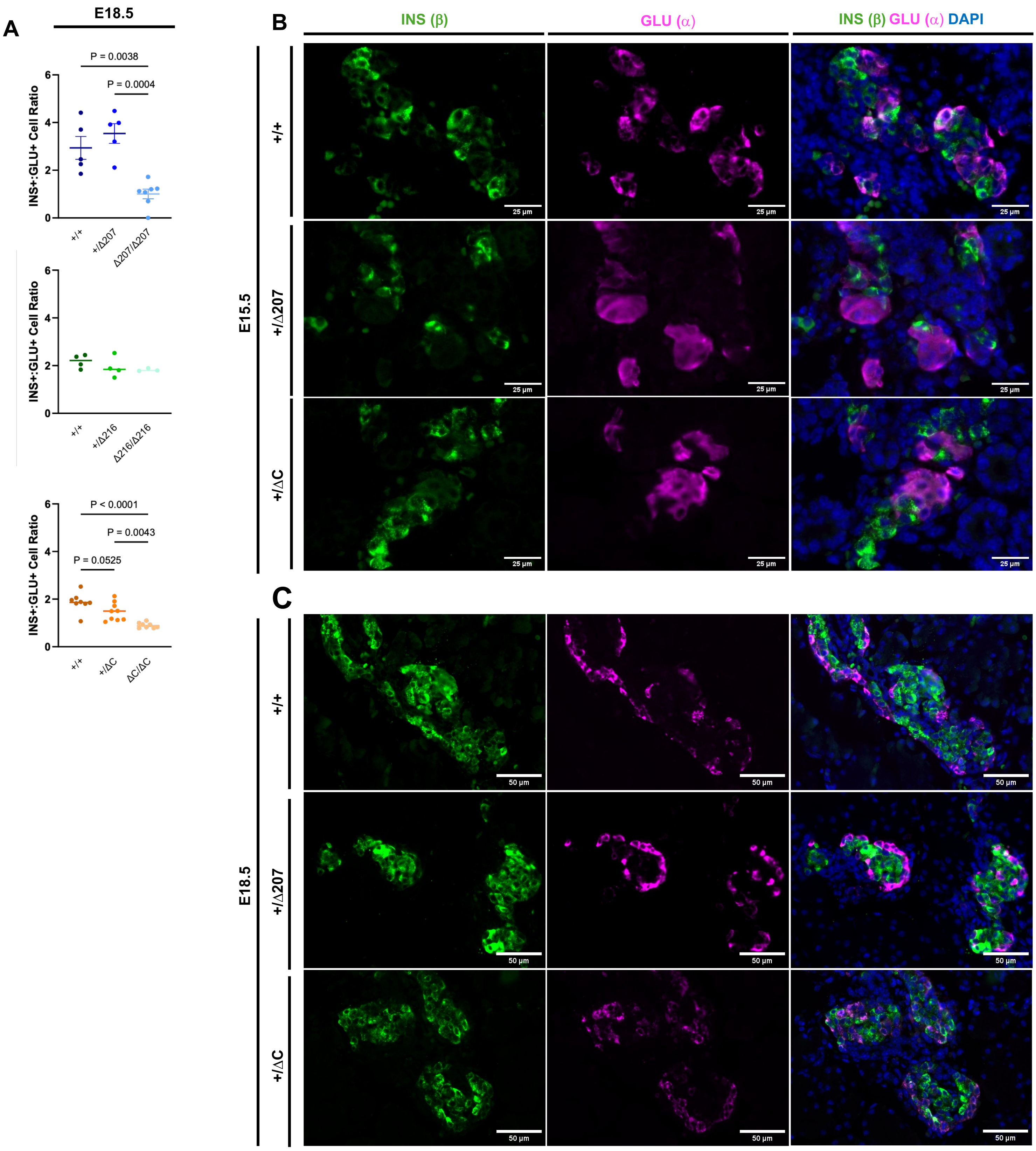

Fig. S4. Pdx1Δ207 heterozygotes have a significant reduction in INS+ cells but not GLU+ cells at E15.5 and E18.5.
(A) INS+:GLU+ cell ratio at E18.5 (n=5-8). (B-C) Tissue sections of pancreata of heterozygotes immunolabeled for INS (green), GLU (magenta), and DAPI (blue) at E15.5 (B) and E18.5 (C). Significant p-values are displayed (one-way ANOVA with Tukey's). Bars indicate mean±s.e.m.

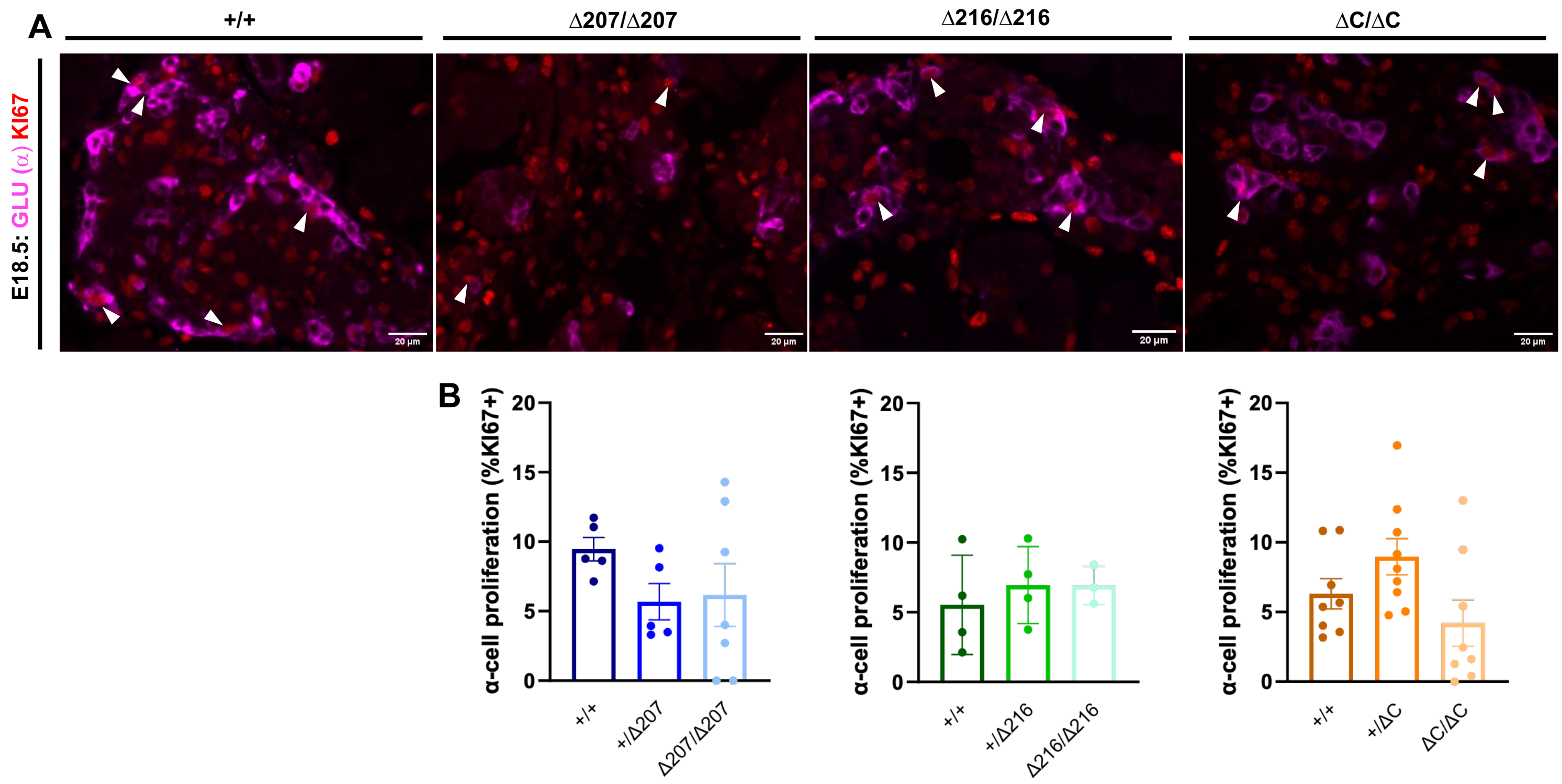

Fig. S5. α-cell proliferation remains unchanged in all PDX1 C-terminal mutants

(A) Tissue sections from E18.5 pancreata immunolabeled for GLU (magenta) and KI67 (red). White arrows point to proliferating α cells. Quantification of α-cell proliferation (%KI67+) at E18.5 (n=5-8). Each dot represents an individual embryo. For each embryo, the mean was calculated from each 3 sections spaced 150 µm apart. Significant p-values are displayed; absence of a value indicates no significant difference (one-way ANOVA with Tukey's). Bars indicate mean±s.e.m.

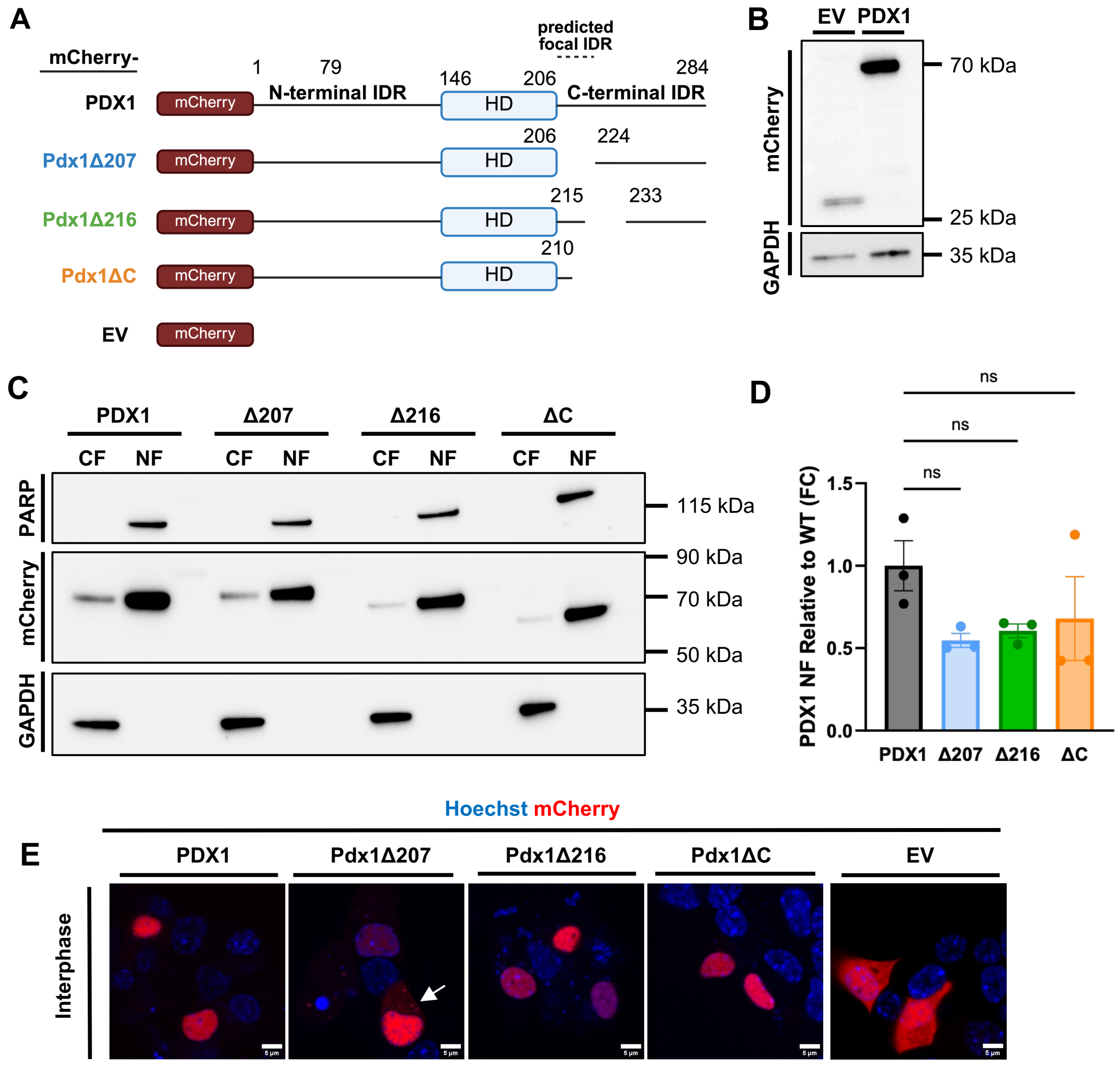

Fig. S6. Deletion of the predicted PDX1 C-terminal focal IDR does not alter nuclear localization

(A) Schematic of N-terminally mCherry tagged PDX1 constructs. Created in BioRender. Wokasch, A., 2026. https://BioRender.com/y2xa37s. (B) Western blot validation of mCherry antibody recognition of mCherry-Pdx1 in the MIN6 β cell line. (C) Subcellular fractionation of mCherry-tagged PDX1 C-terminal mutants PARP represents nuclear fraction (NF). GAPDH represents cytoplasmic fraction (CF) (*n*= 3 biological replicates). (D) Densitometry quantification of PDX1 NF for each construct relative to PARP and normalized to control littermates. (E) Live cell imaging of mCherry-tagged PDX1 C-terminal mutants during interphase. Hoechst labels DNA within nucleus. White arrow represents PDX1 signal within the cytoplasm (n=3 biological replicates with minimum 80+ interphase cells per construct). Significant p-values are displayed; absence of a value indicates no significant difference (one-way ANOVA with Tukey's). Bars indicate mean±s.e.m.

Development • Supplementary information

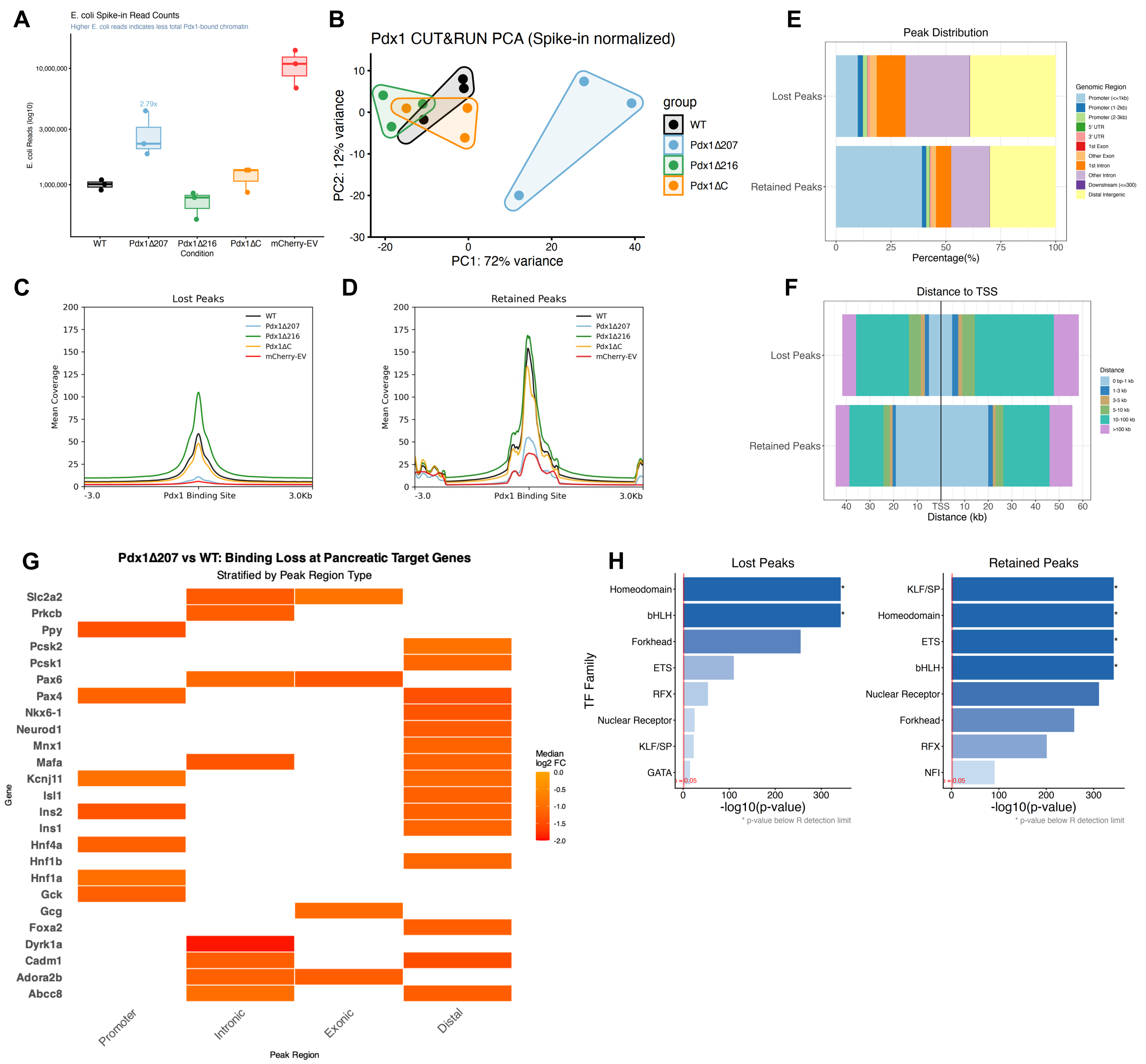

Fig. S7. CUT&RUN-seq spike-in normalization shows globally reduced PDX1 chromatin occupancy with deletion of residues 207-223 (Pdx1Δ207) but not Pdx1Δ216 or Pdx1ΔC

(A) *E. coli* spike-in read counts relative to mouse (mm39) counts per sample across Pdx1 mutant conditions. (n=3). (B) Spike-in normalized principal component analysis (PCA) of CUT&RUN-seq data. (C-D) deepTools3 metagene profile plot of lost (C) and retained (D) peaks for each PDX1 construct. (E) Genomic distribution of Pdx1Δ207 lost peaks annotated using ChIPseeker. (F) Distribution of Pdx1Δ207 lost and retained peaks from the TSS annotated using ChIPseeker. (G) Heatmap displaying median Log2 fold change (Pdx1Δ207 vs wild-type) at key pancreatic target genes stratified by peak region. Each tile represents the median Log2FC for each region colored by intensity for degree of binding loss. (H) HOMER known transcription factor motifs grouped by family, enriched at lost and retained peaks in Pdx1Δ207

Development • Supplementary information

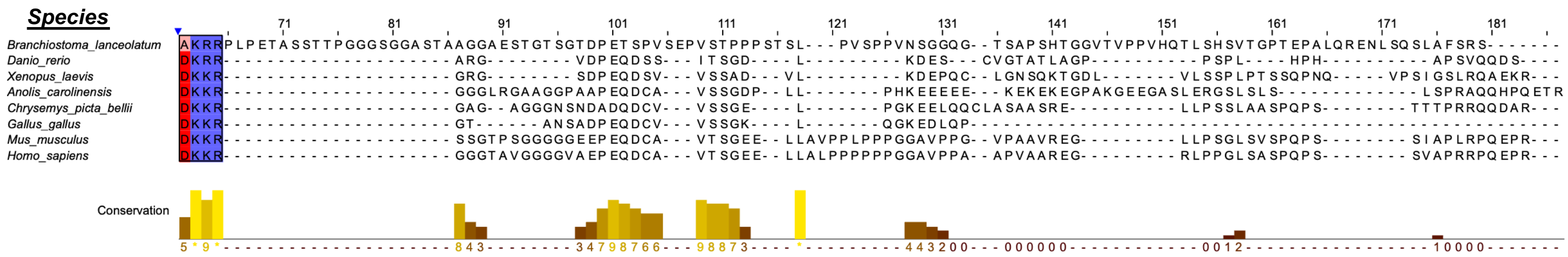

Fig. S8. MSA and sequence conservation of the PDX1 C-terminus (mouse residues 207-284) spanning vertebrates and the invertebrate amphioxus

Table S1. NCBI/UniProt proteins IDs for MSA

Homeodomain TFs (Mouse)	UniProt ID
PDX1	P52946
CDX2	P43241
HOXA1	P09022
HOXA4	P06798
HOXA5	P09021
HOXA6	P09092
HOXA7	P02630
HOXC8	P09025
HOXC12	Q8K5B8
HOXA13	Q62424
HESX1	Q61658
EMX1	Q04742
NKX6-1	Q99MA9
NKX2-2	P42586
PAX4	P32115
PAX6	P63015
LHX2	Q9Z0S2
ISL1	P61372
PO3F2	P31360
PO3F4	P62515
PBX1	P41778
MEIS1	Q60954
**PDX1 Orthologs**	**NCBI/Uniprot ID**
Anolis carolinensis (Anole)	XP_003225569.2
Branchiostoma lanceolatum (Amphioxus)	CAH1267539.1
Chrysemys picta belli (Painted Turtle)	XP_005286710.2
Danio rerio (Zebrafish)	NP_571518.2
Gallus gallus (Chicken)	XP_040514320.1
Homo sapiens (Human)	P52945
Mus musculus (Mouse)	P52946
Xenopus laevis (Xenopus)	NP_001165682.1

Table S2. Antibody dilutions

Reagent			Source	Indentifier
**Primary Antibodies**	**Dilution**	**Antigen Retrival Buffer**		
Rabbit anti-Amylase	IF- 1:500	TEG or Sodium Citrate	Abcam	Cat# 201450
Rabbit anti-ARL13B	IF- 1:200	TEG (pH 9.0)	Proteintech	Cat# 17711-1-AP
Mouse anti-E-cadherin	IF-1:500	TEG (pH 9.0)	BD Tranduction	Cat# 610182
Mouse anti-glucagon	IF- 1:800	Sodium Citrate (pH 6.0)	Millipore	Cat# MABN238
Guinea Pig anti-Insulin	IF- 1:2	Sodium Citrate or TEG	Agilent Dako	Cat# IR00261-2
Rabbit anti-Ki67	IF- 1:500	Sodium Citrate (pH 6.0)	Abcam	Cat# ab15580
Rat anti-Krt19 (TROMA III)	IF- 1:50	TEG (pH 9.0)	DSHB	Cat# TROMA-III, RRID:AB_2133570
Rabbit anti-MafA	IF-1:800	TEG (pH 9.0)	Bethyl/Fortis Life Sciences	Cat# IHC-00352
Rabbit anti-MafB	IF-1:400	TEG (pH 9.0)	Bethyl/Fortis Life Sciences	Cat# A700-046
Armenian hamster anti-MUC1	IF- 1:500	TEG (pH 9.0)	Invitrogen	Cat# MA5-11202
Goat anti-Ngn3	IF- 1:2,000	TEG (pH 9.0)	gift from G. Gu (Vanderbilt)	
Rabbit anti-Pdx1	IF- 1:500	Sodium Citrate (pH 6.0)	Abcam	Cat# ab47267
Goat anti-Pdx1	IF- 1-10,000, WB- 1:5,000	Sodium Citrate (pH 6.0)	gift from C. Wright (Vanderbilt)	
Rabbit anti-pHH3 (Ser-10)	IF- 1:200	Sodium Citrate (pH 6.0)	Millipore	Cat# 06-570
Rabbit anti-Sox9	IF- 1:1,000	TEG (pH 9.0)	Millipore	Cat# AB5535
DAPI	5 mg/ml		Thermo Scientifc	Cat# 62248
Rabbit anti-GAPDH	WB-1:500		Cell Signaling Technology	Cat# 5174S
Rabbit anti-PARP	WB-1:1000		Cell Signaling Technology	Cat#9542S
Rabbit anti-H3K4me3	C&R-0.5 ug		EpiCyper	Cat# 13-0060
Living Colors rabbit anti-DsRed Polyclonal Antibody	WB- 1:1000 C&R- 0.5 ug		Takara	Cat# 6322496
**Secondary Antibodies**				
Alexa Flour® 488-conjugated AffiniPure Donkey Anti-Goat IgG (H+L)	IF: 1:200		Jackson ImmunoResearch	Code: 705-545-147
Cy2-conjugated AffiniPure Donkey Anti-Goat IgG (H+L)	IF: 1:500		Jackson ImmunoResearch	Code: 715-225-150
Alexa Flour® 488-conjugated AffiniPure Donkey Anti-Guinea Pig IgG (H+L)	IF: 1:500		Jackson ImmunoResearch	Code: 706-545-148
Cy3-conjugated AffiniPure Donkey Anti-Goat IgG (H+L)	IF: 1:500		Jackson ImmunoResearch	Code: 705-165-147
Cy3-conjugated AffiniPure Donkey Anti-Rabbit IgG (H+L)	IF: 1:500		Jackson ImmunoResearch	Code: 711-165-152
Alexa Flour® 647-conjugated AffiniPure Donkey Anti-Mouse IgG (H+L)	IF-1:800		Jackson ImmunoResearch	Code: 715-605-150
Cy5-conjugated AffiniPure Donkey Anti-Rabbit IgG (H+L)	IF-1:400		Jackson ImmunoResearch	Code: 711-175-152
Alexa FlourTM 647 goat anti-armenian hamster IgG (H+L)	IF- 1:500		Invitrogen	Cat# A78967
Horseradish Peroxidase (HRP) AffiniPure Donkey Anti-Goat IgG (H+L)	WB-1:7,500		Jackson ImmunoResearch	Code: 705-035-003
Horseradish Peroxidase (HRP)Goat Anti-Rabbit IgG (H+L)	WB-1:7,500		Promega	Cat# W4011

Table S3. Other key reagents

**Western Blot**				
Pierce RIPA Buffer	1X		ThermoScientific	Cat# 89900
Protease Inhibitor Cocktail Tablets	(1:10mls)		Roche	Cat# 05 892 791 001
Phosphatase Inhibitor Cocktail Tablets	(1:10mls)		Roche	Cat# 04 906 845 001
Pierce BSA Protein Assay Kit	N/A		ThermoScientific	Cat# 23225
NuPAGE Sample Reducing Agent (10X)	1X		Invitrogen	Cat# NP0004
NuPAGE LDS Sample Buffer (4X)	1X		Invitrogen	Cat# NP0007
NuPAGE Antioxidant	(1:400)		Invitrogen	Cat# NP0005
NuPage 4%-12% Bis-Tris gel	1.0 mm		Invitrogen	Cat# NP0322BOX
iBlot 2 NC Mini Stacks	N/A		Invitrogen	Cat# IB23002
Restore Western Blot Stripping Buffer	N/A		ThermoScientific	Cat# 21059
Super Signal West Pico Plus Chemiluminescent Substrate	N/A		ThermoScientific	Cat# 34580
**CUT&RUN-seq**				
CUTANA ChIC/CUT&RUN Kit	24rxn		EpiCyper	SKU: 14-1048-24rxn
CUTANA E. coli Spike-in DNA	0.5 ug		EpiCyper	Cat# 18-1401
NEBNext Ultra II DNA Library Kit for Illumina	24rxn		New England Biolabs	Cat# E7645S
NEBNextMultiplex Oligos for Illumnia 96 Unique Dual Index Primer Pairs	96 rxns		New England Biolabs	Cat# E6440S
**Solutions**				
Normal Donkey Serum (NDS)	5%		Jackson ImmunoResearch	Code: 017-000-121
DAKO Antibody Diluent	1X		Agilent DAKO	Cat# S302281
DAKO Protein Block Serum Free	1X		Agilent DAKO	Cat# X090930
Non-fat milk	5%			
Sodium Citrate Antigen Retrival Buffer	1X		N/A	N/A
Tris-EGTA (TEG) Antigen Retrival Buffer	1X		N/A	N/A
Paraformadehyde 16% Solution EM Grade	4%		Electron Microscopy Sciences	Cat# 15710
Triton X-100	0.2%, 1%		Sigma Aldrich	Cat# T8787-250ml
Tween 20	0.10%		Fisher Scientific	Cat# AAJ20605AP
Tris-Buffered Saline (TBS)	1X		N/A	N/A
Ethanol	Various Concentrations		Epredia	Cat# 22-050-106
Methanol	Various Concentrations		Fisher Chemical	
Benzyl Alcohol: Benzyl Benzoate(BABB)	1:2 (Refractive Index 1.55)		N/A	N/A
Xyelnes	1X		Fisher Chemical	Cat# X3P-1GAL
Paraffin	1X		Leica	Cat# 39601006
Eosin	1X		Epredia	Cat# 6766007
Modified Harris Hematoxylin	1X		Epredia	Cat# 72704
VECTASHIELD Vibrance Antifade Mounting Medium	1X		Vector Laboratories	Cat# H-1700-10
CoverSafe	1X		StatLab	Cat# MMC0226

Table S4. PDX1 C-terminal mouse lines

**Mouse Lines**			**Generated/Origin**	**Background**
Pdx1ΔC (aa1-210)			Jackson Laboratory	mixed SV129/C57BL/6J
Pdx1Δ207-223 (aa1-206,224-284)			University of Michigan	C57BL/6J
Pdx1Δ216-232 (aa1-217,233-284)			University of Michigan	C57BL/6J
**CRISPR Generation of Δ207 and Δ216 Mouse Lines**				
Cas9 Protein	30 ng/ul		Sigma	Cat# CAS9PROT
Guide RNA 1 (tagtagcgggaccccgagtg)	30 ng/ul		Synethgo	
Guide RNA 2 (ttgtgcggtgacctcgggcg)	30 ng/ul		Synethgo	
**Oligonucleotides**				
Δ207-223 Forward 'CCGGAGCAAGATTGCGCCGTCACAT'	100 uM		Integrated DNA Technologies	
Δ207-223 Reverse 'GCTAAGGCCCGAAGGCAGTA'	100 uM		Integrated DNA Technologies	
Δ216-232 Forward 'GATAAGAAACGTAGTAGCGGGACCC'	100 uM		Integrated DNA Technologies	
Δ216-232 Reverse 'GCTAAGGCCCGAAGGCAGTA'	100 uM		Integrated DNA Technologies	
ΔC Forward 'AGGAGGTGCTTACACAGCGG '	100 uM		Integrated DNA Technologies	
ΔC Reverse 'GCACAATCTTGCTCCGGCTC'	100 uM		Integrated DNA Technologies	
Transnetyx genotyping probes are propietary information			Transnteyx	

Development • Supplementary information

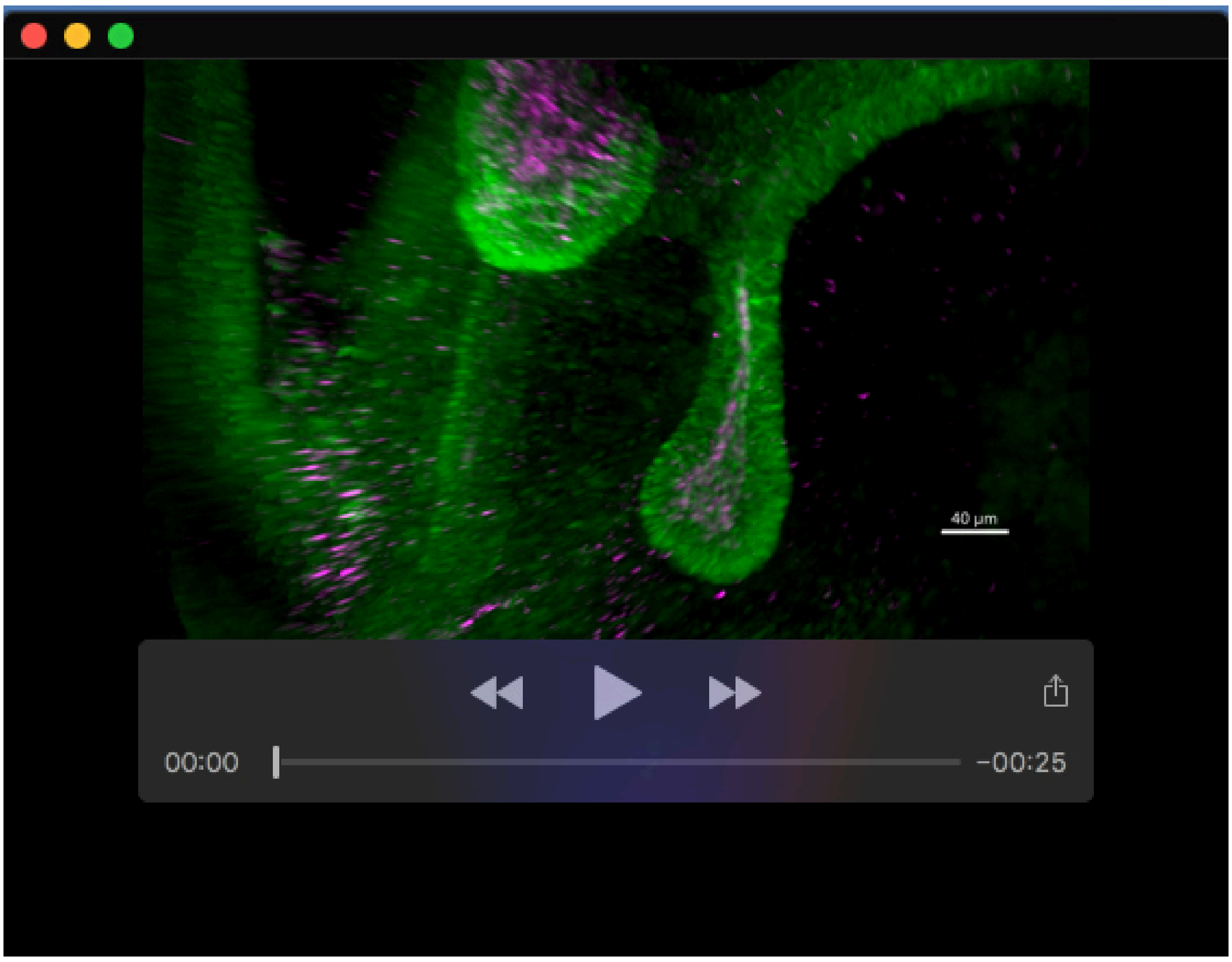

Movie 1. E11.5 Whole-mount wild-type control posterior foregut organs immunolabeled for ECAD (green) and MUC1 (magenta)

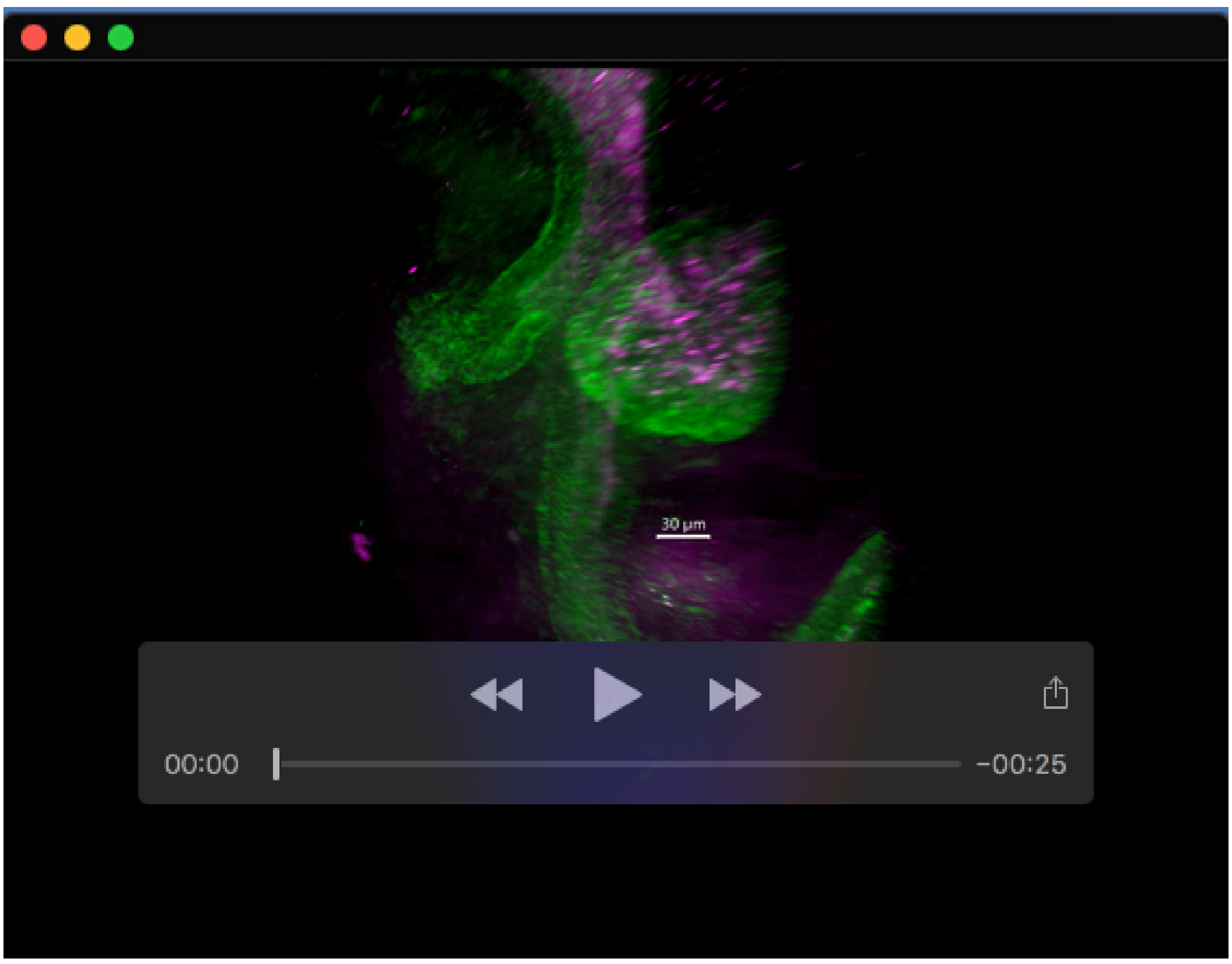

Movie 2. E11.5 Whole-mount Pdx1∆207 homozygote posterior foregut organs immunolabeled for ECAD (green) and MUC1 (magenta)

Development • Supplementary information

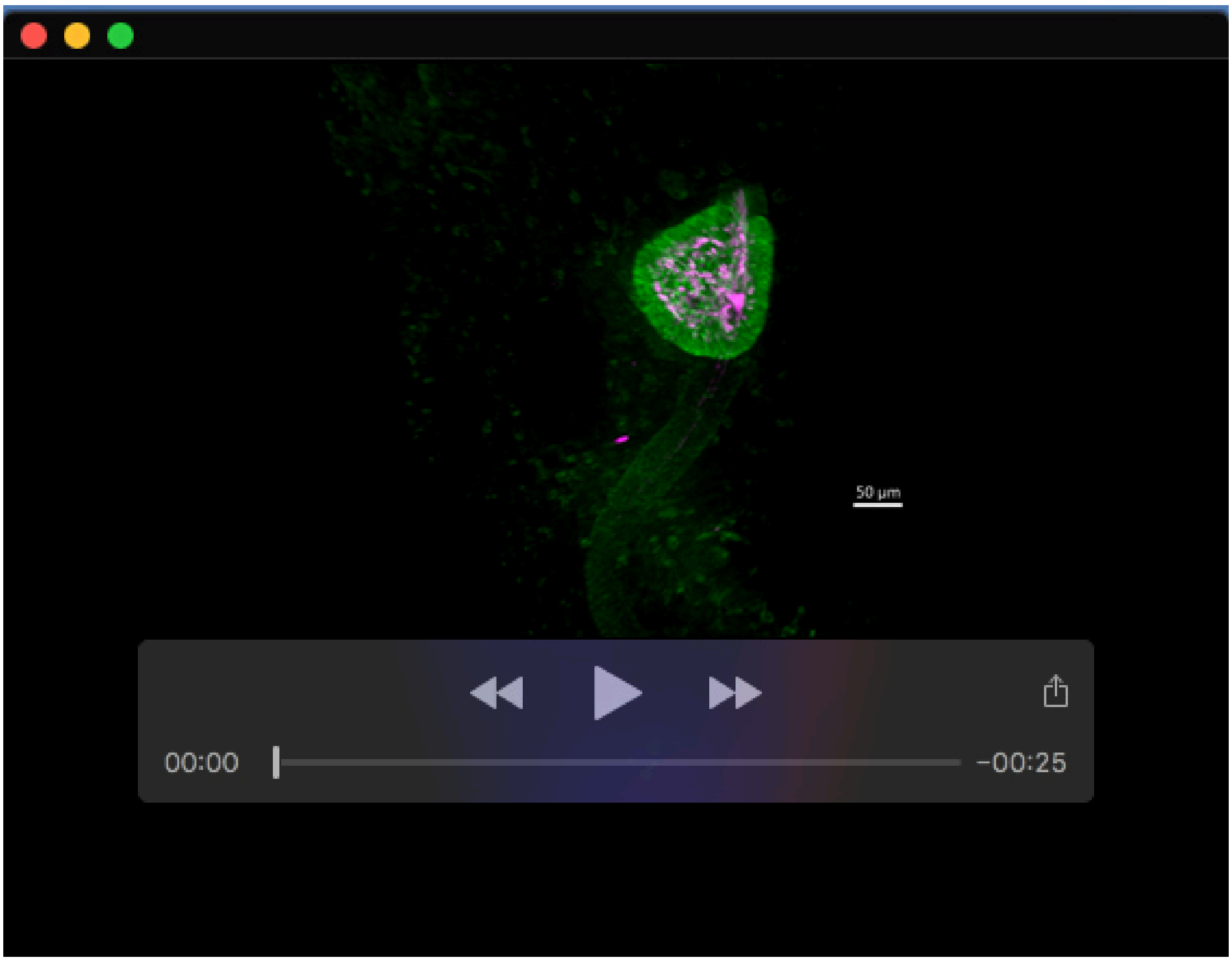

Movie 3. E11.5 Whole-mount Pdx1ΔC homozygote posterior foregut organs immunolabeled for ECAD (green) and MUC1 (magenta)
